# β-Catenin Signaling in Hepatocellular Carcinoma: Mechanisms of Therapeutic Resistance and Emerging Therapeutic Strategies

**DOI:** 10.3390/curroncol33100576

**Published:** 2026-09-25

**Authors:** Anastasia Chambers, Morgan Reeves, Abhishek Shrestha, Siri Yarrapatni, Sergio Duarte, Ali Zarrinpar

**Affiliations:** Department of Surgery, College of Medicine, University of Florida, Gainesville, FL 32608, USA; agchambers@ufl.edu (A.C.); morganelireeves@gmail.com (M.R.); abhishek.shrestha@surgery.ufl.edu (A.S.); s.yarrapatni@ufl.edu (S.Y.); sergio.duarte@surgery.ufl.edu (S.D.)

**Keywords:** hepatocellular carcinoma, β-catenin, Wnt signaling, therapeutic resistance, targeted therapy, immunotherapy, cancer stem cells, tumor microenvironment, immune evasion, precision medicine, liver cancer

## Abstract

Hepatocellular carcinoma (HCC) is a leading cause of cancer-related mortality worldwide, with poor patient outcomes largely attributable to late diagnosis and the frequent development of therapeutic resistance. Although advances in chemotherapy, targeted therapies, immunotherapy, and combination regimens have improved treatment options, durable clinical responses remain limited. This review synthesizes current evidence on the role of β-catenin signaling in HCC progression and therapeutic resistance, emphasizing the molecular mechanisms underlying aberrant pathway activation and its interactions with other oncogenic signaling networks. The review examines how constitutive β-catenin activation promotes tumor growth, cancer stem cell maintenance, metabolic reprogramming, immune evasion, and resistance to chemotherapy, targeted therapies, and immune checkpoint inhibitors. In addition, emerging therapeutic strategies targeting β-catenin are discussed, including inhibition of β-catenin transcriptional activity, modulation of upstream Wnt signaling, direct suppression of *CTNNB1* expression using nucleic acid-based approaches, and interventions targeting the immune and metabolic consequences of pathway activation. Collectively, the evidence identifies β-catenin as a central mediator of HCC progression and treatment resistance while highlighting its potential as a therapeutic target. A deeper understanding of β-catenin-driven mechanisms may facilitate the development of more effective therapeutic strategies and improve clinical outcomes for patients with HCC.

## Simple Summary

Hepatocellular carcinoma (HCC), the predominant type of liver cancer, is one of the leading causes of cancer-related deaths worldwide, and many patients are diagnosed only after the disease has reached an advanced stage. Although new treatments, including targeted therapies and immunotherapy, have improved patient care, many tumors eventually become resistant, limiting long-term treatment success. Increasing evidence has identified β-catenin, a protein involved in normal cell growth and tissue maintenance, as an important contributor to HCC development and resistance to multiple forms of therapy. This review summarizes current knowledge of how abnormal β-catenin signaling promotes tumor growth, supports cancer cell survival, and reduces treatment effectiveness. It also discusses emerging therapeutic strategies designed to inhibit β-catenin signaling or counteract its downstream effects. A better understanding of these mechanisms may guide the development of more effective treatment combinations, improve patient selection for existing therapies, and support future research aimed at overcoming therapeutic resistance in liver cancer.

## 1. Introduction

### 1.1. Liver Cancer Incidence, Mortality, and Complications with Diagnosis

Liver cancer has the sixth highest incidence and third highest cause of cancer mortality globally, making it a significant global health concern. In the year 2022 alone, over 865,000 new cases and 757,000 mortalities were reported worldwide. With the incidence of liver cancer increasing 3-fold over the last four decades, in 2026 an estimated 42,340 new cases will be diagnosed and 30,980 mortalities are expected in the United States alone [1]. Two major histologic types comprise the majority of liver cancer cases: hepatocellular carcinoma (HCC) and cholangiocarcinoma (CCA), with HCC constituting approximately 75–85% of cases globally [2]. HCC is often associated with the presence of an identifiable underlying condition, such as viral infection (hepatitis B or C), alcohol use, and metabolic-dysfunction-associated fatty liver disease (MAFLD) [1,3]. Due to the initially asymptomatic nature of HCC, more than half of patients are diagnosed at an advanced stage, resulting in a five-year survival rate of about 20% [1]. Current methods for detection and diagnosis are limited as the current standard of care biomarker, alpha fetoprotein (AFP), has poor sensitivity (49–61%) [4,5]. Regional therapies, such as surgical resection and radiation therapy, are limited to patients with early stage diagnoses. For patients with advanced-stage disease (Barcelona Clinic Liver Cancer staging system B or C), systemic therapies are the primary treatment option, though these therapies are often life-prolonging rather than curative. Additionally, these systemic regimens are indicated only for patients with well-compensated cirrhosis (Child-Pugh A), as many of the landmark trials that established their efficacy excluded patients with Child-Pugh B or C [6,7].

### 1.2. Current Therapeutic Landscape: Chemotherapy, Targeted Therapy, Immunotherapy, and Combination Treatments

The impaired liver function generally associated with HCC development, activation of autophagy, increased DNA repair mechanisms, and drug efflux transporters renders conventional chemotherapy ineffective due to increased toxicity and reduced sensitivity [8]. As a result, cytotoxic agents such as 5-fluorouracil (5-FU), doxorubicin, and cisplatin play a limited role in systemic treatment [9,10,11]. While no conventional chemotherapy regimen carries FDA approval for systemic treatment of HCC, some of these therapies are used in addition to hepatic arterial infusion therapy (HAIC) and transarterial chemoembolization (TACE) or off-label after patients show progression on approved agents [11,12,13]. This created a need for more specialized treatment, giving way to the development of targeted therapeutics.

Sorafenib, a small molecule inhibitor of multiple tyrosine kinases, was the first FDA approved targeted therapy (2007), and has remained a standard of care for advanced HCC for almost two decades [14]. In recent years, rapid progress in targeted molecular therapies, immunotherapies, and combination treatment strategies has transformed the management of advanced stage HCC [7]. A series of tyrosine kinase inhibitors (TKIs) were developed to inhibit specific molecular abnormalities identified to be involved in HCC progression, especially vascular endothelial growth factor receptor (VEGFR), including lenvatinib, regorafenib, and cabozantinib. Lenvatinib was found to improve overall survival (OS) and progression-free survival (PFS) as a first-line therapy for unresectable HCC compared to sorafenib [15], while second-line therapies regorafenib [16], cabozantinib [17], and ramucirumab (a human IgG monoclonal antibody of VEGFR-2) [18] were found to improve OS and PFS in patients previously treated with sorafenib compared to placebo [7].

The development of immune tolerance in HCC patients, driven by the enrichment of regulatory T cells, myeloid-derived suppressor cells, and changes in key immune checkpoint regulators, led to the implementation of immunotherapies as a potential therapeutic strategy for advanced HCC [19,20]. Two anti-PD-1 (programmed cell death protein-1) monoclonal antibodies have been FDA approved for second-line therapy in HCC, nivolumab and pembrolizumab; while these therapies did not significantly increase OS compared to sorafenib, they had favorable safety profiles and improved OS and PFS compared to placebo [21,22]. Additionally, durvalumab, an anti-programmed cell death protein ligand-1 (PD-L1) monoclonal antibody, showed comparable outcomes to sorafenib.

Beyond single-agent therapies, combination therapeutic strategies have been implemented to enhance clinical efficacy in HCC treatment. Nivolumab has been used in combination with ipilimumab, an anti-cytotoxic T-lymphocyte-associated protein 4 (CTLA-4) monoclonal antibody, as a second-line therapy for patients previously treated with sorafenib; it has demonstrated improved tumor response and increased OS [23]. Recently, nivolumab plus ipilimumab was implemented as a first-line therapy for unresectable HCC and was found to significantly improve OS compared to sorafenib or lenvatinib [24]. Atezolizumab, an anti-PD-L1 mAb, and bevacizumab, an anti-vascular endothelial growth factor (VEGF) mAb, have been used together as a first-line therapy for unresectable HCC; this combination was found to have improved PFS and OS compared to sorafenib [25]. The use of atezolizumab plus bevacizumab as a second-line therapy has been found to have similar positive outcomes [26]. Additionally, atezolizumab has been combined with cabozantinib as a first-line therapy for advanced HCC and had increased PFS compared to sorafenib [27]. Camrelizumab, an anti-PD-L1 mAb, combined with revoceranib, a VEGFR TKI, has demonstrated improved PFS and OS in unresectable HCC as a first-line therapy compared to sorafenib [28]. The combination of durvalumab and tremelimumab, anti-CTLA-4 mAbs, as a first-line therapy in unresectable tumors demonstrated increased OS compared to sorafenib, and had a promising risk–benefit profile in patients exhibiting intolerance or resistance to sorafenib [29,30].

While the use of kinase and immune checkpoint inhibitors has shown a significant extension of survival and successful downstaging, at times leading to increased resection rates, these therapies often face the limitation of development of resistance. This has driven the development of a new field of immunotherapies that focuses on gene targets and advanced techniques such as chimeric antigen receptor (CAR)-T cell [31], dendritic cell vaccine [32], and oncolytic virus therapy [33].

### 1.3. Introduction to β-Catenin: Dual Role in Cell Adhesion and Wnt/β-Catenin Signaling Pathway

β-catenin is an evolutionarily conserved, multifunctional protein, essential in developmental processes and tissue homeostasis, that has two distinct functions within the cell: structure and signaling [34,35,36]. The dual roles of β-catenin are enabled by its structural composition, consisting of 12 Armadillo repeats flanked by flexible N- and C- terminal domains [37,38]. β-catenin serves as a molecular bridge that links cadherin-based adherens junctions to the actin cytoskeleton. The N-terminal domain of β-catenin binds the cytoplasmic tail of classical cadherins, while the central region of Armadillo repeats recruit α-catenin [39,40], which can in turn nucleate F-actin bundles; this stabilizes the cadherin–catenin complex and actin filament network that comprise adherens junctions [36]. The membrane-associated, junction-stabilized fraction of β-catenin is essential for cell–cell adhesion and maintains epithelial integrity. The C-terminal domain of β-catenin can be phosphorylated, modifying its affinity for α-catenin and cadherins, which allows for immediate release of β-catenin from these junctions when signaling cues demand it. Therefore, the loss of β-catenin disrupts junctional adhesion fundamental in cell-cell interactions and tissue architecture [36].

In parallel, β-catenin also acts as a central effector of the canonical Wnt (wingless-related integration site) signaling pathway (Figure 1). In the absence of Wnt, the N-terminal serine/threonine residues of β-catenin are phosphorylated by a destruction complex containing axis inhibition (AXIN), adenomatous polyposis coli (APC), casein kinase 1 (CK-1), and glycogen synthase kinase-3β (GSK3β), marking it for β-transducin repeat-containing protein (β-TrCP)-mediated ubiquitination and proteasomal degradation. In the presence of Wnt, the ligand can bind to Frizzled (FZD) and low-density lipoprotein receptor-related protein 5 and 6 (LRP5/6) receptors resulting in the disassembly of the destruction complex, preventing β-catenin’s degradation. This results in cytoplasmic accumulation of β-catenin and its subsequent translocation to the nucleus (via importin-karyopherin and direct interactions with nucleoporins) where it associates with T cell factor (TCF)/lymphoid enhancer binding factor (LEF) transcription factors [41]. β-catenin’s association with TCF/LEF directs it to Wnt-responsive elements, where it initiates the assembly of a suite of co-activators to drive transcription of genes involved in proliferation, differentiation, and stem-cell maintenance [36].

Alternatively, non-canonical Wnt signaling operates independently of β-catenin. This is carried out through two major branches, the planar cell polarity (PCP) pathway and the Wnt/Ca^2+^ pathway. In PCP signaling, Wnt5a and Wnt11 bind ROR–Frizzled receptor complexes to activate Dvl and Vangl, which drives Rho/Rac1 signaling through ROCK and JNK and leads to cytoskeletal remodeling and transcriptional changes [42]. The Wnt/Ca^2+^ signaling signals through G-protein mediated PLC activation and intracellular calcium flux, engaging kinases such as PKC and CaMKII [43].

Non-canonical and canonical Wnt signaling frequently intersect, often in an antagonistic manner that depends heavily on cellular context. In mammary cancer stem cells, sequestration of Wnt5a and Wnt5b counteracts the inhibitory effect that non-canonical signaling normally exerts on canonical Wnt output, which helps preserve stem cell function [44]. A related dynamic has been described in hepatocellular carcinoma, where non-canonical ligands are preferentially expressed in poorly differentiated tumor cells, and Wnt5a directly suppresses β-catenin/TCF-driven canonical signaling in these cells [45]. These findings point to a tissue-specific interplay between the two arms of Wnt signaling that likely shifts depending on tumor stage and differentiation state.

The Wnt/β-catenin pathway plays a critical role in development and homeostasis of a range of tissues, and dysregulation of this signaling pathway can result in a wide range of ailments, such as heart, lung, liver, bone, and nervous system diseases [46]. In addition to all these diseases, β-catenin signaling is frequently dysregulated in cancers and is known to be dysregulated in 50% of HCC cases. The aim of this paper is to investigate the role β-catenin plays in HCC and development of resistance to therapeutic intervention [47].

## 2. β-Catenin Signaling in Hepatocellular Carcinoma

### 2.1. Aberrant β-Catenin Activation in HCC: Mechanism and Origin/Existence and Relevance

As the central effector of the Wnt/β-catenin signaling pathway, β-catenin’s stability and subcellular localization are normally under strict regulatory control. In cancer, the canonical Wnt/β-catenin pathway can become constitutively “on” and thus signals independently of those oscillating signals seen in healthy tissues [48]. Aberrant activation has been documented across multiple cancer types, including colorectal, gastrointestinal, glioblastoma, lung, breast, melanoma and HCC [49,50,51,52,53]. This highlights β-catenin as a broadly conserved oncogenic pathway, although its biological consequences are highly context-dependent. In the liver, β-catenin regulates hepatocyte proliferation, metabolic zonation, regeneration after injury, and immune tolerance. Dysregulated β-catenin signaling in HCC thus gives rise to tumor phenotypes distinct from those observed in other Wnt-driven cancers [54].

In HCC, β-catenin activation occurs through diverse genetic and non-genetic mechanisms that ultimately promote accumulation and transcriptional activity of the active, non-phosphorylated protein (Figure 2). The most common genetic alterations are gain-of-function mutations in the *CTNNB1* gene [55,56], predominantly within exon 3, which encodes serine and threonine residues required for phosphorylation by CK-1 and GSK3β. Mutation of these residues renders β-catenin resistant to degradation, leading to its cytoplasmic accumulation and subsequent nuclear translocation where it transcriptionally reprograms the cell [57]. Alternatively, loss-of-function mutations in components of the destruction complex impair β-catenin phosphorylation and turnover [58].

Beyond genetic alterations, β-catenin signaling can also be enhanced through mechanisms such as increased ligand availability, epigenetic silencing of pathway inhibitors, and microenvironmental cues associated with chronic inflammation and fibrosis. Increased expression of Wnt ligands and Frizzled receptors increases pathway activation in HCC [59,60]. Multiple microRNAs and long non-coding RNAs have been shown to regulate β-catenin stability, nuclear localization, and downstream transcriptional activity, further contributing to pathway dysregulation [61]. Importantly, these findings suggest that β-catenin activation extends beyond *CTNNB1* mutation status alone and may occur through diverse regulatory mechanisms.

Disruption of the membrane-associated pool of β-catenin can further modulate pathway activity [62,63]. In some tumors, in particular well-differentiated epithelial HCCs, cytoplasmic and nuclear β-catenin accumulate due to impaired degradation while membrane-associated β-catenin at adherens junctions remain largely preserved. In others, loss or destabilization of E-cadherin during epithelial–mesenchymal transition weakens adherens junctions, releasing β-catenin from the plasma membrane and increasing the intracellular pool available for transcriptional activity [64,65]. These cases emphasize the distinction between the two biological pools.

#### Prevalence of β-Catenin Mutations/Activation in HCC Patient Cohorts

Large patient cohort studies have demonstrated that *CTNNB1* mutations are reported in roughly 30–50% of HCC cases, although prevalence varies according to patient population, underlying liver disease etiology, and molecular classification methodology. Mutations in AXIN1, a scaffolding protein of the β-catenin destruction complex, are observed in approximately 5–10% of HCCs [66], whereas mutations in destruction complex component APC, are rare (<5%) and unlike in colorectal cancer where they have a high frequency [67]. However, as mentioned before, mutation status underestimates the overall frequency of β-catenin signaling in HCC. Consistent with this concept, 40–60% of HCC cases showed no mutations in *CTNNB1* or AXIN1/2, which led to a genome-wide association study revealing alcohol-related HCC to be specifically linked to *WNT3A-WNT9A* variants. Furthermore, evidence shows mutations in Wnt ligands, FZD receptors, and secreted frizzled-related proteins (SFRPs) are seen in 95% of HCC and 68% of surrounding tumor tissues [59,68,69,70]. One cohort from China assessed for mRNA expression of β-catenin exon 3 mutation showed 44.1% of HCC cases and 61.8% showed increased expression in the cytoplasm or nucleus [71]. *CTNNB1* mutations have also been shown to be more frequent in Asian populations and in older patients and can occur in livers without cirrhosis [72]. These mutations are enriched in alcohol-related, Hepatitis C virus (HCV)-associated, and metabolic dysfunction-associated steatotic liver disease (MASLD)-driven HCC, while Hepatitis B virus (HBV)-associated tumors tend to harbor *CTNNB1* mutations less frequently [73].

The timing and origin of β-catenin activation during hepatocarcinogenesis remain under active investigation. Evidence suggests that β-catenin dysregulation may function as either an early or late event depending on disease context and accompanying molecular alterations. In some settings, β-catenin activation appears to contribute to early clonal expansion and tumor initiation, while in others it may emerge during tumor progression as part of broader oncogenic reprogramming. Metabolic dysfunction, such as type 2 diabetes mellitus, may further exacerbate β-catenin-driven tumorigenesis through extracellular matrix remodeling and enhanced signaling. Environmental exposures, including aflatoxin, have also been linked to mutations in Wnt pathway scaffolding genes like AXIN1 and APC, illustrating the interplay between genetics and environment in shaping tumor biology. Consequently, functional pathway activation, rather than mutation prevalence, may more accurately identify tumors in which β-catenin contributes to tumor progression and therapeutic resistance.

### 2.2. Consequences of Constitutive β-Catenin Activation Phenotype and Function/Biological Consequence

Constitutive β-catenin signaling contributes to multiple stages of HCC development, influencing tumor differentiation state, cellular behavior, metabolic adaptation, stemness-associated phenotypes, and interactions with the tumor microenvironment (Figure 2). Although β-catenin activation is frequently associated with differentiated tumors, persistent pathway activation promotes oncogenic transcriptional programs supporting tumor growth, survival, and therapeutic resistance. Furthermore, the biologic output of β-catenin signaling is heavily influenced by accompanying molecular alterations and microenvironmental context, contributing to the heterogeneity observed among β-catenin-active HCCs.

#### 2.2.1. Effects on Hepatic Differentiation and Tumor Phenotype

In normal liver tissue, β-catenin contributes to hepatic zonation and regulates genes involved in ammonia detoxification, glutamine synthesis, and bile acid metabolism. Consequently, constitutive activation of this pathway in HCC often preserves aspects of hepatocyte identity despite malignant transformation. A characteristic of constitutive β-catenin activation in HCC is a differentiated hepatocyte-like tumor phenotype. *CTNNB1*-mutated tumors frequently demonstrate pseudoglandular architecture with intracellular or canalicular bile accumulation [74]. Diffuse glutamine synthetase expression is observed and commonly used as a surrogate marker of β-catenin pathway activation in clinical pathology [75,76]. These tumors often display lower AFP expression relative to poorly differentiated progenitor-like HCC subclasses. In addition to differentiated hepatocyte features, β-catenin-active HCCs are increasingly recognized as a distinct immune-excluded molecular subclass [77,78,79]. Molecular classification studies consistently demonstrate reduced inflammatory signaling and diminished immune cell infiltration in *CTNNB1*-mutated tumors. This propensity towards an immune-cold phenotype further distinguishes β-catenin-active HCC from inflammatory or progenitor-like subclasses and has important implications for therapeutic responsiveness.

#### 2.2.2. Effects on Tumor Cell Behavior

Persistent β-catenin sustains a proliferative milieu that facilitates continued tumor expansion; as it translocates to the nucleus, it functions as a transcriptional co-activator inducing the expression of cell cycle progression genes such as cyclin D1 and myelocytomatosis (MYC). β-catenin signaling contributes to tumor cell survival under stressful microenvironmental conditions. Studies have demonstrated that β-catenin activation suppresses apoptotic signaling and enhances resistance to cellular stress, thereby promoting tumor persistence during chronic liver injury and hepatocarcinogenesis [80]. Cooperative interactions with additional oncogenic pathways, including phosphoinositide 3-kinase (PI3K)/protein kinase B (AKT) and mesenchymal–epithelial transition factor (MET) signaling, further amplify these survival effects and contribute to aggressive tumor growth in experimental models.

b-catenin signaling-mediated metabolic reprogramming such as altered glucose utilization, glutamine metabolism, lipid metabolism, and oxidative stress responses may provide selective growth advantages within metabolically stressed hepatic environments [81]. These effects appear particularly relevant in metabolic dysfunction-associated steatotic liver disease and metabolically associated steatohepatitis (MASH)-associated hepatocarcinogenesis, where nutrient stress and lipid dysregulation contribute substantially to tumor development.

Despite some β-catenin-active tumors demonstrating differentiated hepatocyte-like morphology, studies show that β-catenin signaling may also contribute to migration, invasion, and epithelial–mesenchymal transition (EMT) [82,83]. These effects appear highly context-dependent and are often mediated through cooperation with additional signaling pathways including transforming growth factor-β (TGF-β), yes-associated protein (YAP), and PI3K/AKT. In these settings, β-catenin signaling regulates transcriptional programs associated with motility and extracellular matrix remodeling. These findings highlight the importance of signaling context in determining whether β-catenin activation primarily supports differentiated hepatocyte programs or more aggressive mesenchymal phenotypes.

#### 2.2.3. Stemness, Plasticity and Dedifferentiation

β-catenin-driven transcription supports a progenitor-like phenotype [84,85,86,87], enabling tumor initiation and plasticity, contributing to tumor heterogeneity, therapeutic resistance, and recurrence. The Wnt/β-catenin signaling is vital in maintaining cancer stem cells (CSCs) and tumor initiating cells (TICs). Studies have demonstrated that β-catenin activation in HCC may increase sphere-forming ability, tumor-initiating capacity, and expression of stemness markers including epithelial cell adhesion molecule (EpCAM) and prominin-1 (CD133) [88]. Non-coding RNAs further modulate β-catenin-mediated stemness and plasticity programs at the post-transcriptional level, allowing tumor cells to dynamically transition between differentiated and stem-like states. Oncogenic miRNAs, including miR-130a [89], miR-1246 [90], and miR-5188 [91], can enhance β-catenin signaling and thus increase stemness, EMT, and resistance. Likewise, lncRNAs such as lncTCF7 sustain liver cancer stem cells and promote tumor progression [92]. Conversely, tumor suppressive miRNAs such as miR-34a [93,94] and miR-200 [95] family members attenuate β-catenin activity, reducing self-renewal and invasive capacity.

The influence of these regulatory networks illustrates that stemness-associated phenotypes in HCC arise not from isolated β-catenin activation alone, but rather through extensive pathway integration and transcriptional reprogramming. Cancer stem cells are characterized by enhanced resistance to chemotherapy, targeted therapies, and immune-mediated elimination. β-catenin signaling has been implicated in resistance to sorafenib and immune checkpoint blockade, potentially through maintenance of stemness-associated programs and immune exclusion phenotypes [96,97,98,99].

#### 2.2.4. Tumor Microenvironment and Immune Consequences

In addition to its direct effects on tumor cells, constitutive β-catenin activation influences the tumor microenvironment and immune landscape of HCC. One of the most clinically significant observations in recent HCC literature is the strong association between β-catenin activation and immune exclusion [100]. *CTNNB1*-mutated tumors frequently demonstrate reduced infiltration of cytotoxic T cells, impaired dendritic cell recruitment, and diminished antitumor immune responses, resulting in an immune-cold tumor phenotype.

Mechanistically, β-catenin signaling alters cytokine and chemokine networks involved in immune cell recruitment and activation. Impaired dendritic cell infiltration may subsequently reduce T-cell priming and antitumor immune surveillance. Molecular classification studies consistently identify β-catenin-active HCCs as a non-inflammatory tumor subtype, further supporting the concept that β-catenin signaling contributes to immune evasion during hepatocarcinogenesis [101]. Other pivotal studies highlight that β-catenin activation is associated with reduced responsiveness to immune checkpoint blockade [101,102,103,104]. As immune checkpoint inhibitors assume increasingly central roles in advanced HCC management, understanding the mechanisms underlying β-catenin-associated immune exclusion has become a major area of preclinical and translational research.

Beyond immune interactions, β-catenin signaling is also influenced by and contributes to broader microenvironmental processes including fibrosis, hypoxia, and stromal remodeling. Most HCCs arise in chronically injured and fibrotic livers, where persistent inflammatory signaling, extracellular matrix remodeling, and cytokine production shape tumor behavior. Crosstalk between β-catenin signaling and pathways associated with fibrosis and hypoxia may further support angiogenesis, metabolic adaptation, and tumor progression within the chronically diseased hepatic microenvironment [105,106].

### 2.3. Crosstalk with Other Oncogenic Pathways

β-catenin signaling in HCC does not operate in isolation. Extensive crosstalk with other oncogenic and inflammatory pathways shapes tumor behavior and therapeutic vulnerabilities. Such signaling integration contributes substantially to the molecular heterogeneity observed among β-catenin-active HCCs.

#### 2.3.1. Cooperative

Experimental models consistently demonstrate that activated β-catenin synergizes with additional oncogenic drivers. Co-activation with c-MET signaling drives hepatocyte proliferation, tumor initiation, and invasive behavior [107,108]. Mechanistically, c-MET signaling converges on PI3K/AKT and mitogen-activated protein kinase (MAPK), key pathways regulating cellular proliferation and survival while simultaneously reinforcing β-catenin transcriptional activity.

Integration with the PI3K/AKT/mammalian target of rapamycin (mTOR) axis represents an important node of crosstalk. AKT-mediated phosphorylation and inhibition of GSK3β reduces β-catenin degradation, facilitating its stabilization and nuclear accumulation [48]. β-catenin also interfaces with additional kinase-driven networks, including rat sarcoma (RAS)/rapid accelerated fibrosarcoma (RAF)/MAPK signaling, through shared upstream growth factor receptors and inflammatory mediators.

Crosstalk between β-catenin and Hippo signaling has also emerged as an important driver of HCC progression. Loss of Hippo pathway activity results in YAP activation, which cooperates with β-catenin to promote proliferation, stemness-associated phenotypes, and tumor growth.

#### 2.3.2. Context-Dependent

The effects of β-catenin are influenced by the metabolic, inflammatory, and immune context in which signaling occurs. This context dependence contributes substantially to the phenotypic heterogeneity observed among β-catenin-active HCCs. Integration with metabolic and hepatocyte lineage-regulating networks can be seen through interactions with transcriptional regulators such as hepatocyte nuclear factor 4-alpha (HNF4α) and peroxisome proliferator-activated receptor (PPAR) family members [109,110,111]. Nuclear factor erythroid 2-related factor 2 (NRF2) crosstalk is associated with enhanced metabolic plasticity, antioxidant systems, and tumor cell survival under conditions of chronic hepatic injury [112]. Redox balance may influence pathway activity, as elevated reactive oxygen species (ROS) levels can suppress Wnt/β-catenin signaling through degradation of key pathway components [113], whereas ROS-limiting mechanisms support β-catenin-dependent stemness programs [114,115].

Hypoxic conditions common within advanced tumors enhance β-catenin transcriptional activity through hypoxia-inducible factor-1α (HIF-1α)-dependent mechanisms [116], while inflammatory signaling pathways including interleukin-6 (IL-6)/Janus kinase (JAK)/signal transducer and activator of transcription 3 (STAT3) and nuclear factor kappa-light-chain-enhancer of activated B cells (NF-κB) can augment Wnt signaling and promote expression of β-catenin target genes [117,118,119]. These interactions reinforce proliferative and survival programs and contribute to epithelial–mesenchymal transition, stemness-associated phenotypes, and therapeutic adaptation.

β-catenin participates in bidirectional communication with the tumor microenvironment. Tumor-associated macrophages, particularly M2-polarized macrophages, secrete factors such as Tumor Necrosis Factor-α (TNF-α) that promote β-catenin activation, epithelial–mesenchymal transition, and cancer stem cell maintenance [120]. As touched on earlier, activated β-catenin remodels the immune microenvironment by suppressing chemokine programs required for dendritic-cell recruitment and effective T-cell priming.

β-catenin signaling is further shaped by non-immune stromal elements. Hepatic stellate cells (HSCs), the principal source of cancer-associated fibroblasts (CAFs) in liver tumors, become activated in response to chronic injury and secrete TGF-β, PDGF, and other fibrogenic mediators that both remodel the extracellular matrix (ECM) and modulate Wnt/β-catenin signaling in adjacent tumor cells [121,122]. Hepatocyte–HSC crosstalk in HCC is bidirectional, with β-catenin representing one signaling axis through which this reciprocal relationship can be mediated [123]. ECM remodeling driven by CAFs and HSCs alters matrix stiffness and integrin signaling, which can further potentiate β-catenin nuclear localization [124]. β-catenin-active tumors are also characterized by altered angiogenic programs, with endothelial cell interactions and VEGF-related signaling contributing to vascular remodeling in the tumor microenvironment, complementing the HIF-1α-driven effects on β-catenin activity described above [106,125].

## 3. Mechanisms Linking β-Catenin to Drug Resistance

### 3.1. Generalized Mechanisms of Resistance

Activation of the Wnt/β-catenin signaling pathway, most commonly through *CTNNB1* and AXIN1 mutations and other pathway perturbations, predominates in HCC. These alterations drive β-catenin accumulation and its nuclear translocation, where it complexes with TCF/LEF transcription factors to upregulate pro-survival and proliferative genes that drive tumorigenesis. β-catenin signaling is also a key contributor to both innate and acquired resistance against therapeutic regimens. One central mechanism underlying this drug resistance is β-catenin-driven enrichment of CSCs [126], bolstered by epigenetic silencing of β-catenin suppressors [127] and a self-sustaining ephrin type-B receptor 2 (EPHB2)/proto-oncogene tyrosine-protein kinase Src (SRC)/AKT/GSK3β positive feedback loop. Additionally, a diverse set of upstream activators converges on β-catenin to promote resistance. Drug efflux transporters, such as ATP-Binding Cassette (ABC) transporters, are known for mediating resistance in HCC by reducing the intracellular accumulation of therapeutic agents. β-catenin acts as an upstream mediator of ABC transporters, and inhibition of β-catenin signaling was found to enhance drug sensitivity via transporter up-regulation [128]. Collectively, these mechanisms (stemness induction, EMT promotion, crosstalk, and drug-efflux activation) position β-catenin as a central driver of resistance to many conventional therapeutics in HCC.

### 3.2. Chemotherapy Resistance

In the clinic, hepatocellular carcinoma frequently resists chemotherapy despite intensive dosing regimens. This paradox is driven by subtle molecular rewiring that shields cancer cells from cytotoxic insults, and a persistently hyperactive β-catenin signaling pathway sits at its core in HCC, endowing tumor cells with stem-like traits, metabolic resilience, and anti-apoptotic defenses that render most cytotoxic agents ineffective.

Pharmacological activation of β-catenin via 6-bromoindirubin-3′-oxime, a GSK3β inhibitor, was found to reduce growth inhibition of interferon-α/5-fluorouracil (IFN-α/5-FU) combination therapy more than 50% in HCC cells and was able to restore DNA synthesis. Nuclear accumulation of β-catenin drives expression of cancer stem cell markers such as EpCAM, which was found to be a marker of non-response. These findings suggest that activation of the Wnt/β-catenin signaling pathway induces chemoresistance to IFN-α/5-FU therapy [129]. Furthermore, 5-FU itself has been found to amplify β-catenin signaling, in turn enhancing stemness and resulting in chemotherapeutic response.

Prolonged exposure to anthracyclines, such as doxorubicin, results in acquired resistance in HCC. The β-catenin/TCF transcription complex upregulates target genes zinc finger E-box binding homeobox 1 (*ZEB1*), *c-myc*, and *cyclin D1*, promoting epithelial–mesenchymal transition in HCC cells. The disruption of the interaction between β-catenin and TCF and subsequent reduced expression of β-catenin target genes, resensitized HCC cells to doxorubicin, suggesting Wnt/β-catenin signaling plays a critical role in this resistance development [130].

Patients with cisplatin exposure and in turn acquired cisplatin resistance had elevated miR-130a levels. There is evidence that miR-130a directly inhibits the expression of runt-related transcription factor 3 (RUNX3), a known tumor suppressor gene, which subsequently resulted in the activation of Wnt/β-catenin signaling and increased drug resistance, suggesting cisplatin resistance is reinforced by β-catenin-linked pathways [89].

### 3.3. Targeted Therapy Resistance

Approximately 30% of HCC patients are responsive to tyrosine kinase inhibitors, the primary class of targeted therapies approved for advanced HCC, and the majority of those cases develop resistance within six months of treatment [131]. Wnt/β-catenin signaling plays a central role in mediating resistance to these TKIs; activated β-catenin has been directly associated with sorafenib and regorafenib resistance in HCC patients, and emerging evidence implicates the Wnt/β-catenin signaling pathway in lenvatinib and cabozantinib resistance.

#### 3.3.1. Sorafenib

Sorafenib is the most well-established example of β-catenin-linked TKI resistance in HCC. Patient-derived ex vivo drug screening and subsequent tissue analysis found higher nuclear β-catenin to be associated with worse outcomes and reduced sorafenib response. These findings were further validated in vitro across a panel of HCC cell lines, demonstrating that nuclear β-catenin levels correlated with sorafenib IC50 values. Inhibition of Wnt/β-catenin signaling, particularly by disrupting β-catenin’s interaction with CREB-binding protein (CBP), increased apoptosis and restored sorafenib sensitivity [132]. Mechanistically, sorafenib resistance has been repeatedly linked to the persistence of CSCs. Leucine-rich repeat-containing G-protein-coupled receptor 5 (LGR5)-positive CSCs express high levels of lysine-specific demethylase 1 (LSD1/KDM1A), which inhibits the expression of multiple β-catenin suppressors, and were designated as sorafenib resistant, and inhibition of KDM1A reduced β-catenin activity and re-sensitized the cells to sorafenib [127]. An additional recurrent mechanism of β-catenin resistance is a positive feedback loop centered on EPHB2. In HCC patient-derived xenografts resistant to sorafenib, EPHB2 is highly expressed. EPHB2 activates the SRC/AKT/GSK3β/β-catenin signaling pathway and is upregulated by TCF1, in turn establishing a positive feedback loop with the Wnt/β-catenin signaling pathway [126]. Silencing of EPHB2 using short hairpin RNA resensitized HCC tumors to sorafenib. Other mediators of sorafenib resistance also converge on the same signaling pathway. Prospero-related homeobox 1 (PROX1) promotes sorafenib resistance by enhancing β-catenin transcription and subsequent nuclear translocation [133]. Similarly, NIMA-related kinase 2 (Nek2) and low-density lipoprotein receptor-related protein 8 (LRP8) induce β-catenin activation and contribute to sorafenib resistance [134,135].

#### 3.3.2. Regorafenib

While evidence for regorafenib is not as robust, findings indicate that β-catenin is implicated in intrinsic regorafenib resistance but may not play a role in acquired or long-term resistance. Karabicici et al. demonstrated this in vitro in HCC cell line Huh7, where treatment of naïve cells with regorafenib activated Wnt/β-catenin signaling and increased stem/progenitor markers. Furthermore, treatment with Wnt3a/R-Spondin-1 (R-Spo1), activators of the canonical Wnt/β-catenin pathway, mitigated the effect of regorafenib-induced apoptosis. In contrast, long-term regorafenib treatment to establish a resistant cell line resulted in diminished Wnt/β-catenin activity and enhanced TGF-β signaling in its place. Together this evidence suggests that β-catenin mediates early stage stemness-linked resistance to regorafenib, whereas long-term acquired regorafenib resistance may transition to dependence on a TGF-β-driven EMT-like state [136]. Recent studies have shown that DNA Topoisomerase II alpha (TOP2A) inhibition is capable of reversing regorafenib resistance in HCC cells and connect the effects to the Wnt/β-catenin pathway [137].

#### 3.3.3. Levantinib

β-catenin has been found to contribute to stemness-linked levantinib resistance; however, this was specific to a subset of HCC cases rather than an all-encompassing mechanism of resistance. Guo et al. demonstrated that interferon regulatory Factor 2 (IRF2) promotes proliferation, reduces apoptosis, and increases levantinib resistance in HCC by regulating β-catenin expression [138]. The role of β-catenin in resistance was further validated in a more stem cell-focused HCC model, where FZD10 upregulation via methyltransferase-like 3 (METTL3)-dependent m6A was responsible for activating β-catenin and YAP1, driving tumorgenicity and self-renewal. The conclusion was that the FZD10/β-catenin/c-Jun/mitogen-activated protein kinase (MEK)/extracellular signal-regulated kinase (ERK) axis determines the response of HCC cells to levantinib treatment, and inhibition of FZD10 or β-catenin restored levantinib sensitivity in multiple preclinical resistance models [139]. Clinical validation in a cohort of HCC patients treated with levantinib plus nivolumab reported no statistically significant difference in tumor response rate in patients with *CTNNB1* mutation, suggesting that, while β-catenin may be a driver of resistance in specific HCC subsets, it cannot be used as a stand-alone predictor of levantinib response [140].

#### 3.3.4. Cabozantinib

While cabozantinib, a second line TKI that acts as a dual MET and VEGFR2 inhibitor, lacks any direct links to β-catenin-induced resistance, the literature indicates a potential link in MET/β-catenin-driven HCC. An in vivo murine HCC model co-expressing c-MET and ΔN90-β-catenin found that cabozantinib monotherapy did not reduce tumor burden; however, cotreatment with cabozantinib and MLN0128, a dual mTOR inhibitor that targets activated β-catenin effectors, led to reduced tumor burden [141]. Wnt/β-catenin activation often interacts with a second oncogenic signaling pathway rather than acting alone, and in the context of cabozantinib that could mean β-catenin could act as a resistance-enabling player that preserves proliferation and survival signaling rather than directly driving resistance.

### 3.4. Immunotherapy Resistance

The stabilization and activation of β-catenin allows HCC tumors to undergo transcriptional changes that drive immune evasion [101]. The most striking mechanistic consequence of these β-catenin-mediated changes is the suppression of chemokines. The absence of these signals transforms the tumor microenvironment from an inflamed “hot” niche, to a “cold” zone unable to recruit dendritic cells and where effector CD8+ T cells are unable to infiltrate [142]. Additionally, β-catenin can also downregulate the molecular machinery of exosome biogenesis such as Ras-related protein Rab-27A (RAB27A) and syndecan-4 (SDC4) [143], limiting the spread of tumor-related danger-associated molecular patterns that would otherwise heighten the local immune microenvironment. Simultaneously, the transcriptional repression of interferon regulatory factor 2 (IRF2) and POU Class 2 homeobox 1 (POU2F1) can erode type I/II interferon signaling, depriving T cells of a molecular “on-switch” that drives their clonal expansion [144]. Collectively, these pathway-level effects create a tumor-intrinsic barrier that undermines a wide spectrum of immunotherapy strategies.

#### 3.4.1. Anti-PD1 Therapy

The most well-established example of β-catenin-induced immunotherapy resistance involves anti-PD1 immune checkpoint inhibitors (ICIs). A preclinical mouse HCC model demonstrated that β-catenin-driven tumors were resistant to anti-PD1 therapy, while their wild-type β-catenin tumors remained responsive [101]. Mechanistically, this resistance was traced to defective dendritic cell recruitment and impaired T cell activity due to β-catenin inhibition of chemokine C-C motif ligand 5 (CCL5) expression [142]. This is functionally significant because this response creates a tumor state that is intrinsically less responsive to the checkpoint blockade. In a syngeneic mouse HCC model, the use of RNAi trigger DCR-BCAT, which targets *CTNNB1*, significantly increased T cell infiltration into the tumor microenvironment and lead to enhanced tumor sensitivity to anti-PD1 therapy [145]. Similarly, preclinical studies using delivery of β-catenin small interfering RNA (siRNA) [146] and CBP/β-catenin inhibitor (ICG-001) [104] in combination with anti-PD1 therapy showed reduced tumor burden. Clinically, prospective genotyping of patients treated with anti-PD1 or anti-PDL1 therapies found that patients with *CTNNB1* or AXIN1 mutation experienced progressive disease and significantly shorter progression-free survival [147]. In a separate clinical trial for an anti-PD1 monoclonal antibody, nivolumab, two of the three patients with *CTNNB1* mutations were non-responsive [148]. These clinical findings have motivated the use of β-catenin-targeted therapies to restore anti-PD1 sensitivity. A clinical trial combining the transducin β-like protein (TBL)/β-catenin inhibitor tegavivint with pembrolizumab (NCT05797805) is currently ongoing.

#### 3.4.2. Anti-PDL1 Therapy

Evidence demonstrating the role of β-catenin in resistance to anti-PDL1 therapy is less direct as clinically it is rarely used as a monotherapy in HCC. Anti-PDL1 therapies are often administered in combination with anti-VEGF therapy, such as atezolizumab (anti-PDL1) plus bevacizumab (anti-VEGF). The IMbrave150 phase III clinical trial established this combination as first-line standard of care with a response rate of about 30%, indicating that some mechanism of innate resistance may exist [25,149]. Recent findings indicate that β-catenin activation contributes to this primary resistance. A lipid nanoparticle-encapsulated siRNA targeting *CTNNB1* was found to cause alteration in both innate and adaptive immunity β-catenin suppression, resulting in an enhanced ICI response via the prevention of T cell exhaustion and lymphoid aggregate formation. In the IMbrave150 cohort, *CTNNB1* mutational status was inversely correlated with expression of lymphoid aggregate gene signatures, which are prognostic for survival [144]. Interestingly, a clinical case report described a patient with multicentric HCC whose β-catenin-positive nodules progressed on atezolizumab plus bevacizumab, while the β-catenin-negative nodules responded. Additional molecular profiling confirmed that β-catenin expression correlated with ICI resistance at the individual lesion level [150]. This was further validated in a larger HCC patient cohort where β-catenin activation was inversely correlated with CD8+ T cell infiltration and PDL1 expression, and that PD-L1-negative tumors exhibited *CTNNB1* mutations at a higher frequency (35%) than PD-L1-high tumors (21%) [151].

#### 3.4.3. CTLA-4 Blockade and Combined Checkpoint Inhibitor Therapy

CTLA-4 blockade has also been used for therapeutic intervention in HCC, especially tremelimumab-based regimens; however, to date the literature describing the role of β-catenin in CTLA-4 blockade therapy resistance is limited. The proposed mechanism is that Wnt/β-catenin driven immune exclusion likely limits the broader class of checkpoint inhibitors, like CTLA-4 blockade, not just the PD1/PD-L1 blockade. Similarly, in combination immunotherapy, if β-catenin suppresses dendritic cell recruitment and T cell infiltration, then the addition of a second immune checkpoint inhibitor may only modestly improve efficacy, unless the immune exclusion state is able to be reversed. These findings further highlight the increasing need to use *CTNNB1* status as a potential exclusion biomarker for ICI therapy in HCC.

## 4. Therapeutic Strategies Targeting β-Catenin in HCC

Given the growing body of evidence on the critical role of β-catenin activation in HCC progression, therapeutic resistance, immune exclusion, and metabolic reprogramming, several strategies have been developed to intervene in the pathway at different levels. These approaches include disruption of β-catenin transcriptional activity, inhibition of upstream Wnt ligand–receptor signaling, stabilization of the β-catenin destruction complex, and direct suppression of *CTNNB1* expression using nucleic acid-based therapies [48]. While many of these strategies remain preclinical, they provide an important framework for understanding how β-catenin-driven HCC may be therapeutically targeted, particularly in combination with TKIs or immune checkpoint inhibitors (Figure 3).

The major therapeutic strategies targeting Wnt/β-catenin signaling, together with their mechanisms of action, potential advantages, translational limitations, and current stage of preclinical or clinical development, are summarized in Table 1.

### 4.1. Targeting β-Catenin Transcriptional Activity

#### 4.1.1. β-Catenin-TCF/LEF Disruption/Inhibition

Early attempts to therapeutically inhibit β-catenin focused on blocking its interaction with TCF/LEF, the core transcriptional complex responsible for canonical Wnt target-gene activation. Small-molecule inhibitors such as PKF115-854 and CGP049090 were identified through high-throughput screening and shown to disrupt β-catenin/TCF binding, thereby suppressing β-catenin-dependent transcriptional programs [152]. In HCC models, these compounds demonstrated dose-dependent antiproliferative and cytotoxic effects. However, their clinical translation has been limited by modest potency, off-target effects, and unfavorable pharmacokinetic properties. Despite these limitations, β-catenin/TCF-disrupting compounds established proof of principle that transcriptional β-catenin activity is druggable and provided the conceptual basis for more selective approaches.

A newer direct approach is FOG-001 (zolucatetide), which disrupts the β-catenin–TCF interaction and is currently undergoing phase I/II evaluation in Wnt-pathway-activated solid tumors, including an HCC cohort [153]. Clinical activity and the therapeutic window in HCC remain under investigation.

#### 4.1.2. β-Catenin-CBP Inhibition

A more refined transcriptional strategy involves disrupting the interaction between β-catenin and CBP, a coactivator that supports β-catenin-driven oncogenic transcription. ICG-001 and its clinical derivative PRI-724 selectively inhibit β-catenin/CBP binding without directly disrupting β-catenin/TCF interactions [132]. This distinction is important because CBP-associated β-catenin signaling has been linked to stemness, proliferation, and therapeutic resistance. In preclinical HCC models, PRI-724 suppresses expression of oncogenic β-catenin transcriptional targets, including c-Myc and survivin, and enhances sorafenib-induced apoptosis. These findings are consistent with the resistance mechanisms discussed above, where nuclear β-catenin activity and cancer stem cell persistence contribute to reduced TKI sensitivity. Although early phase clinical studies of PRI-724 have shown acceptable safety, monotherapy activity has been limited, suggesting that CBP inhibition is more likely to be effective as part of rational combination therapy rather than as a stand-alone treatment.

#### 4.1.3. β-Catenin/CBP-Directed Modulation

E7386 represents a more recent orally administered approach to β-catenin-directed transcriptional modulation. In its first-in-human study, monotherapy demonstrated limited antitumor activity, including no objective responses in the *CTNNB1*-mutant HCC expansion cohort, although disease stabilization was observed in a subset of patients [154]. Subsequent clinical development has therefore emphasized combination strategies, particularly with lenvatinib and immune checkpoint blockade. Early translational studies of E7386 plus lenvatinib in HCC have demonstrated tumor regression in a subset of patients and pharmacodynamic evidence of treatment-associated transcriptional and antiangiogenic effects; however, the small cohort size and absence of comparative data preclude definitive conclusions regarding clinical efficacy. Additional studies combining E7386 with pembrolizumab, with or without lenvatinib, have been completed, although mature HCC-specific peer-reviewed efficacy data remain limited.

#### 4.1.4. BCL9 Cofactor Inhibition

More recently, B-cell lymphoma 9 (BCL9) has emerged as another therapeutically relevant co-transcriptional regulator of β-catenin. BCL9 facilitates full β-catenin transcriptional activity and appears particularly important in linking β-catenin activation to immune suppression in HCC. Aberrant β-catenin signaling can repress IRF2- and POU2F1-dependent interferon responses, reduce antigen presentation, and promote immune exclusion. BCL9 further contributes to an immunosuppressive tumor microenvironment by supporting tumor-associated macrophage dysfunction through pathways such as bone morphogenetic protein 4 (BMP4) and cluster of differentiation 24 (CD24)–sialic acid-binding Ig-like lectin 10 (SIGLEC10) [155]. Pharmacologic inhibition of BCL9 using the cell-penetrating peptide hsBCL9Z96 has been shown to reprogram tumor-associated macrophages toward a more inflammatory M1-like phenotype, restore antigen presentation, and increase CD8^+^ T-cell infiltration. In vivo, BCL9 inhibition synergizes with anti-PD-L1 therapy, supporting the concept that β-catenin transcriptional blockade may be useful not only for direct tumor suppression but also for reversing immune checkpoint inhibitor resistance. However, further optimization of peptide stability, delivery, biomarker selection, and HCC subtype specificity will be required before this approach can be translated clinically.

#### 4.1.5. TBL1/β-Catenin Inhibition

Tegavivint represents another downstream strategy and interferes with β-catenin transcriptional activity through TBL1. Early phase I/II evaluation in advanced HCC has provided preliminary evidence of activity, particularly in tumors harboring Wnt-pathway alterations; these findings are discussed further in the context of biomarker selection below [156].

### 4.2. Targeting Upstream Wnt Signaling and β-Catenin Stability

#### 4.2.1. Extracellular Wnt Blockade

Targeting β-catenin activation upstream, either by blocking Wnt ligand–receptor engagement or by promoting β-catenin degradation, offers another direct way to halt the signaling cascade that fuels HCC [157]. Extracellular Wnt blockade includes neutralizing antibodies against Wnt ligands and monoclonal antibodies against Frizzled receptors. For example, anti-Wnt2 antibodies can reduce ligand-driven Wnt signaling, while Frizzled-directed antibodies such as OMP-18R5/vantictumab prevent Wnt ligands from engaging their receptors [158]. These approaches are most relevant in tumors where β-catenin activation remains ligand-dependent. However, their utility in HCC has been constrained by pathway redundancy, toxicity, and a narrow therapeutic window, reflecting the physiologic importance of Wnt signaling in tissue homeostasis and regeneration.

#### 4.2.2. PORCN Inhibition

Small-molecule inhibitors of Wnt secretion and β-catenin stabilization have also been explored. Porcupine O-acyltransferase (PORCN) inhibitors such as LGK-974/WNT-974 and ETC-159 block Wnt ligand palmitoylation and secretion, thereby reducing upstream Wnt pathway activation. These agents may be useful in ligand-dependent Wnt-driven tumors and have been shown to enhance radiosensitivity in selected HCC models. However, their activity is expected to be limited in *CTNNB1*-mutant HCC, where β-catenin activation occurs downstream of ligand stimulation and is therefore relatively independent of Wnt secretion [159]. Similarly, tankyrase inhibitors such as XAV939, G007-LK, and JW55 stabilize AXIN and promote β-catenin degradation by reinforcing the destruction complex [160]. While effective in ligand-driven models, tankyrase inhibition is less likely to overcome mutation-driven β-catenin activation, again emphasizing the importance of genotype and pathway context when selecting Wnt-targeted strategies.

Clinical evaluation of PORCN inhibition has also included ETC-159 (ETC-1922159), an orally administered inhibitor of Wnt ligand palmitoylation and secretion. In a first-in-human phase I study, ETC-159 produced dose-dependent pharmacodynamic suppression of Wnt signaling, including reductions in AXIN2 expression; however, no objective responses were observed, although prolonged stable disease occurred in a small number of patients [161]. Treatment was also associated with increased β-CTX and reductions in bone mineral density in some patients, consistent with the on-target skeletal effects of systemic Wnt inhibition [162]. Subsequent phase Ib evaluation of ETC-159 combined with pembrolizumab demonstrated clinical benefit in a subset of patients, including one partial response and five cases of stable disease among 15 evaluable patients, while maintaining evidence of pathway inhibition through AXIN2 suppression [163]. These findings support continued investigation of PORCN inhibition in combination strategies, although the biological utility of this approach is expected to remain greatest in tumors that retain ligand-dependent Wnt signaling rather than those driven by downstream *CTNNB1* or APC alterations [161].

#### 4.2.3. CXCR6 Inhibition

Recent studies have shown that the overexpression and hyperactivation of C-X-C chemokine receptor type 6 (CXCR6) and its ligand C-X-C chemokine ligand 16 (CXCL16) are implicated in HCC progression and β-catenin stabilization [164,165,166]. CXCR6-knockdown in HCC cell lines resulted in reduced p38 activity, decreased Ser-9 phosphorylation of GSK3β, and diminished β-catenin accumulation [164]. A small-molecule CXCR6 antagonist, SBI-457, was found to have favorable pharmacokinetics and antitumor activity in a murine HCC xenograft model [167]. In a follow-up study, SBI-457 attenuated sorafenib induced β-catenin upregulation and nuclear accumulation, suggesting CXCR6 antagonism via SBI-457 can modulate β-catenin activation and could be useful target for upstream regulation of Wnt/β-catenin signaling [166].

#### 4.2.4. Indirect/Repurposed Pathway Modulation

Several clinically available or naturally derived compounds have also been reported to modulate Wnt/β-catenin signaling. Nonsteroidal anti-inflammatory drugs, including aspirin, indomethacin [168], sulindac [169], celecoxib [170], and related agents, may exert indirect Wnt-inhibitory effects through cyclooxygenase (COX)-dependent and COX-independent mechanisms. However, their antitumor activity in HCC has generally been modest and has not established a validated therapeutic role. Natural compounds such as astaxanthin have attracted interest because of their broader effects on oxidative stress, metabolism, apoptosis, and β-catenin degradation. Recent preclinical work suggests that astaxanthin can suppress HCC proliferation by promoting β-catenin ubiquitination and degradation through inhibition of the deubiquitinase USP39 [171]. Although these compounds are unlikely to replace direct pathway inhibitors, they highlight the possibility that β-catenin activity may be therapeutically influenced through broader metabolic and proteostatic mechanisms.

Clinical experience with upstream Wnt inhibition has reinforced concerns regarding the therapeutic window of this strategy. Vantictumab, a monoclonal antibody targeting multiple Frizzled receptors, was evaluated in combination regimens in pancreatic [172] and breast cancers [173], where evidence of antitumor activity was accompanied by clinically significant skeletal toxicity, including fragility fractures that ultimately limited further clinical development. Ipafricept (OMP-54F28), a soluble FZD8-Fc decoy receptor that sequesters Wnt ligands, similarly entered early phase solid-tumor studies, in which fragility fractures and changes in bone-resorption markers were observed at higher exposures [174]. Ipafricept was also evaluated with sorafenib in a completed phase Ib HCC trial (NCT02069145); however, results have not been posted in the trial registry, and we identified no peer-reviewed efficacy report from this HCC study [175].

### 4.3. Direct CTNNB1 Suppression Using Nucleic Acid-Based Therapeutics

#### 4.3.1. Antisense-Mediated CTNNB1 Suppression

Because many β-catenin-activated HCCs harbor downstream *CTNNB1* mutations, direct suppression of β-catenin expression may offer a more precise strategy than upstream ligand or receptor blockade. Early RNA interference approaches targeting Wnt ligands have demonstrated measurable in vitro effects but were limited by delivery challenges and insufficient in vivo validation [176]. Locked nucleic acid antisense oligonucleotides directed against *CTNNB1* showed greater promise, particularly in *CTNNB1*-mutant mouse models, where β-catenin suppression reduced tumor growth. However, these effects appear genotype-dependent, with more limited activity in β-catenin–wild-type tumors [177].

#### 4.3.2. RNA Interference

More recent lipid nanoparticle-formulated siRNA approaches have substantially strengthened the therapeutic rationale for direct *CTNNB1* targeting. Lipid nanoparticle (LNP)-encapsulated siRNA targeting *CTNNB1* has shown robust antitumor activity in *CTNNB1*-mutant HCC models, producing marked tumor regression and broader tumor reprogramming. β-catenin knockdown shifts pericentral, zone 3-like tumor cells toward a more differentiated zone 1/2 hepatocyte-like phenotype, suggesting that *CTNNB1* suppression can remodel the metabolic identity of β-catenin-driven tumors rather than simply inhibit proliferation. In parallel, *CTNNB1* knockdown restores IRF2- and POU2F1-dependent interferon signaling, enhances antigen presentation, promotes CD8^+^ T-cell infiltration, and reverses immune exclusion. These immune effects are particularly important given the association between β-catenin activation and resistance to checkpoint blockade. In preclinical models, LNP-*CTNNB1* also synergizes with immune checkpoint inhibitors, providing a strong rationale for combining direct β-catenin suppression with immunotherapy in biomarker-selected HCC patients [144].

### 4.4. Targeting Immune and Metabolic Consequences of β-Catenin Activation

The therapeutic importance of β-catenin inhibition in HCC extends beyond direct suppression of tumor-cell proliferation. β-catenin activation also shapes the tumor microenvironment by coordinating immune exclusion and lineage-associated metabolic programming, two features that are increasingly recognized as major determinants of therapeutic resistance. In β-catenin-activated HCC, suppression of IRF2- and POU2F1-dependent interferon signaling reduces antigen-presentation capacity and limits effective CD8^+^ T-cell recruitment. At the same time, activation of macrophage-directed immune-evasion programs, including the CD24–SIGLEC10 axis, can impair tumor-associated macrophage phagocytosis and reinforce an immunosuppressive microenvironment. These mechanisms provide a biological basis for the clinical and preclinical observation that β-catenin-activated tumors often behave as immunologically “cold” tumors with reduced sensitivity to immune checkpoint blockade [144].

#### 4.4.1. Immune Reprogramming

This immune phenotype also helps explain why different β-catenin-targeted approaches may have therapeutic value beyond their immediate effects on β-catenin transcription. Direct *CTNNB1* suppression, CBP inhibition, and BCL9 blockade have each been shown in preclinical settings to interfere with β-catenin-dependent immune suppression. Rather than simply reducing oncogenic Wnt output, these interventions may restore interferon responsiveness, improve antigen presentation, increase CD8^+^ T-cell infiltration, and shift tumor-associated macrophages toward a more inflammatory, M1-like state. In this context, β-catenin inhibition may convert a tumor microenvironment that is poorly responsive to immune checkpoint inhibitors into one that is more permissive to antitumor immunity. This provides a strong rationale for combining β-catenin-directed therapies with anti-PD-1 or anti-PD-L1 strategies in biomarker-selected HCC.

#### 4.4.2. Metabolic Reprogramming

In parallel, β-catenin activation maintains zonation-dependent metabolic programs that contribute to tumor identity and therapeutic vulnerability. *CTNNB1*-mutant HCCs frequently retain features of pericentral, zone 3-like hepatocyte biology, including metabolic dependencies related to glutamine utilization, xenobiotic metabolism, and other β-catenin-regulated liver zonation programs. Therapeutic suppression of β-catenin can reprogram these tumors toward a more differentiated zone 1/2 hepatocyte-like state, suggesting that β-catenin inhibition does not merely block growth signaling but can also remodel the metabolic state of the tumor [178]. This concept is particularly important because metabolic specialization may influence sensitivity to TKIs, immune checkpoint blockade, and emerging metabolic therapies.

These observations support therapeutic strategies that exploit both the tumor-intrinsic and microenvironmental consequences of β-catenin inhibition. Reversal of immune exclusion and disruption of β-catenin-dependent metabolic programs provide a rationale for combination approaches incorporating immune checkpoint blockade, TKIs, or metabolic interventions. Their clinical utility, however, will depend on accurate identification of β-catenin-dependent tumors and the ability to achieve sufficient pathway inhibition while preserving physiologic Wnt/β-catenin functions.

### 4.5. Biomarker-Guided β-Catenin-Directed Therapy

Across mutational categories, evidence for clinical usefulness of biomarkers varies between mechanistic support and true clinical validation. Nuclear β-catenin, GS staining, and *CTNNB1*/Wnt-pathway mutation tumors define an immune-excluded “cold” phenotype that shows reduced response to checkpoint inhibitors [101,104]. Retrospective human cohorts show enrichment of *CTNNB1* mutations in a treatment-resistant “immune-exclusion” class [179]. The molecular heterogeneity of HCC represents an important consideration in the development of β-catenin-directed therapies. Wnt/β-catenin pathway activation may arise through *CTNNB1* gain-of-function mutations, alterations in AXIN1 or APC, increased Wnt ligand–receptor signaling, or non-genetic mechanisms. Consequently, the therapeutic level at which the pathway is targeted should ideally reflect the underlying mechanism of pathway activation. Upstream approaches, including PORCN or Frizzled inhibition, would be expected to have limited efficacy in tumors harboring constitutively active *CTNNB1*, whereas direct β-catenin suppression or inhibition of nuclear transcriptional cofactors may remain relevant in this molecular context.

#### 4.5.1. Nuclear β-Catenin Accumulation and Glutamine Synthetase Expression

Genomic alterations alone, however, may not fully identify tumors with functional β-catenin activation. Nuclear β-catenin accumulation and diffuse glutamine synthetase (GS) expression are commonly used as tissue-based surrogates of Wnt/β-catenin pathway activation in HCC. Although clinicopathologic studies have demonstrated associations among *CTNNB1* mutations, nuclear β-catenin localization, and GS expression, the concordance between diffuse GS staining and exon 3 *CTNNB1* mutation is incomplete [180]. Tissue-based markers may therefore complement, rather than substitute for molecular profiling.

#### 4.5.2. Genome Profiling

*CTNNB1* and other Wnt-pathway alterations therefore represent candidate biomarkers for molecular enrichment in clinical trials. Prospective genomic profiling of HCC demonstrated an association between activating Wnt/β-catenin alterations and primary resistance to immune checkpoint blockade. Patients with pathway alterations had a disease-control rate of 0%, compared with 53% among patients with Wnt-wild-type tumors, and a median progression-free survival of 2.0 versus 7.4 months [147]. These findings support Wnt-pathway alterations as a candidate for negative predictive biomarkers of immunotherapy response while providing a rationale for evaluating β-catenin-directed therapies in this molecular subgroup.

Early clinical experience with tegavivint, a downstream inhibitor of Wnt/β-catenin, further supports a biomarker-enriched approach. In an ongoing trial of advanced HCC, Wnt-pathway alterations were assessed using liquid biopsy, and clinical activity was predominantly observed among patients harboring pathway alterations, whereas substantial benefit was not observed in patients without detectable Wnt-pathway alterations [156]. Although confirmation in larger prospective cohorts is required, these observations suggest that alterations involving *CTNNB1*, AXIN1, APC, and related pathway components may have potential utility as predictive biomarkers for downstream β-catenin inhibition rather than serving solely as indicators of pathway activation. Additional functional biomarkers, including AXIN2 expression, β-catenin target-gene signatures, circulating tumor DNA, and pharmacodynamic alterations in downstream transcriptional targets, may provide further evidence of pathway activity and therapeutic target engagement.

#### 4.5.3. Molecular Profiling and Emerging Techniques

Recent advancements in high-resolution molecular profiling techniques beyond the traditional bulk genomic and transcriptomic approaches have provided new avenues to investigate the complexity of β-catenin-driven HCC tumors. Single-cell RNA sequencing and spatial transcriptomic/proteomic approaches provide the ability to address heterogeneity within these tumors and evaluate how β-catenin activations affect cell populations within these tumor environments. Integration of these techniques has identified distinct *CTNNB1*-mutant hepatocyte populations and their response to β-catenin suppression. This highlights the role of β-catenin signaling in HCC at a cellular and spatial level and its effect on the TME and the immune-excluded phenotype often observed in this subset of HCC tumors [144,181,182]. This multi-omic approach provides a clearer classification of β-catenin-driven tumors as a singular technique may not capture the complete picture of the functional state of β-catenin signaling within the tumor [183].

Furthermore, CRISPR-based functional screenings can use known associations from molecular profiling and identify specific genes or pathways that are functionally required for proliferation or therapeutic resistance [184,185]. Finally, the use of artificial intelligence (AI)-based approaches allows for more complex and robust datasets of these techniques to be evaluated to identify clinically meaningful subcategorizations of tumors expressing β-catenin. Collectively, these approaches shift the field from defining β-catenin-driven HCC by a singular genetic alteration to a more comprehensive functional classification, allowing for more personalized treatment strategies.

#### 4.5.4. The Non-Invasive Biomarker, EOB-MRI

In addition to these biopsy-based biomarkers, gadolinium ethoxybenzyl diethylenetriaminepentaacetic acid-enhanced magnetic resonance imaging (Gd-EOB-DTPA-MRI: EOB-MRI) could provide a noninvasive technique for identifying HCC cases with activated Wnt/β-catenin signaling [186]. During the hepatobiliary phase of HCC, the uptake of gadolinium ethoxybenzyl diethylenetriaminepentaacetic acid (gadoxetic acid) is primarily mediated by the hepatocyte organic anion transporting polypeptide 1B3 (OATP1B3). While the majority of HCC cases demonstrate reduced OATP1B3 expression and appear hypointense, tumors with activated Wnt/β-catenin signaling often exhibit an iso- or hyperintense appearance due to an increase in OATP1B3 expression [186,187]. Even though not every Wnt/β-catenin-active tumor will have the same imaging phenotype, this association between OATP1B3 expression and Wnt/β-catenin pathway activation could be useful in the determination of therapeutic selection due to these tumors’ increased likelihood of having an immune-excluded TME and reduced response to ICIs. EOB-MRI have been implemented in identification of Wnt/β-catenin-active tumors; however, they are not a definitive substitute for molecular testing [188].

An effective biomarker-guided strategy may therefore require integration of tumor genotype, tissue phenotype, and functional pathway activity rather than reliance on any single marker. Such an approach could distinguish ligand-dependent tumors potentially amenable to upstream Wnt inhibition from mutation-driven tumors requiring downstream β-catenin blockade. It may also identify β-catenin-active, immune-excluded tumors in which combination with immune checkpoint inhibition is biologically rational. Prospective biomarker-enriched studies will be required to determine whether these molecular and phenotypic features have predictive utility independent of their prognostic associations.

### 4.6. Clinical Translation and Limitations of Systemic Wnt/β-Catenin Inhibition

Despite encouraging preclinical findings, clinical translation of Wnt/β-catenin-directed therapies has been limited by the essential physiologic functions of this pathway in normal adult tissues. Wnt signaling contributes to intestinal epithelial renewal, bone remodeling, hematopoiesis, and tissue regeneration. Consequently, systemic pathway inhibition may produce mechanism-based toxicities that reflect disruption of normal Wnt-dependent homeostasis rather than nonspecific drug effects. This limitation is particularly relevant to upstream inhibitors that suppress ligand-dependent signaling in both malignant and nonmalignant tissues. The clinical development, efficacy signals, and principal translational limitations of representative Wnt/β-catenin-directed agents are summarized in Table 2.

#### 4.6.1. Frizzled Receptor and Wnt Ligand Inhibition Limitations

Clinical experience with upstream Wnt inhibition has reinforced concerns regarding the therapeutic window of this strategy. Vantictumab [172] and ipafricept [173] have illustrated the challenge of achieving therapeutically meaningful inhibition of ligand-dependent Wnt signaling while preserving the physiological Wnt activity required for skeletal homeostasis. Skeletal toxicity has been among the most consistently observed adverse effects of systemic Wnt inhibition. In the first-in-human study of the Frizzled-8 decoy receptor ipafricept, increased markers of bone resorption and fragility fractures were observed at higher drug exposures. Similar concerns emerged during clinical evaluation of the anti-Frizzled antibody vantictumab, prompting incorporation of bone-turnover monitoring, dual-energy X-ray absorptiometry, and prophylactic or therapeutic bisphosphonate administration into subsequent protocols. Although such mitigation strategies may reduce fracture risk, bone toxicity contributed to limitations in the clinical development of ipafricept. These observations illustrate the difficulty of achieving sustained antitumor Wnt inhibition without perturbing normal Wnt-dependent skeletal homeostasis [174].

#### 4.6.2. PORCN Inhibition Limitations

Clinical studies of PORCN inhibition have similarly demonstrated a disconnect between pharmacodynamic pathway suppression and antitumor efficacy. In a phase I study of WNT974 involving 94 patients with advanced solid tumors, reduced AXIN2 expression in skin and tumor biopsies provided evidence of pathway inhibition; however, no objective responses according to RECIST criteria were observed, and stable disease was achieved in only 16% of patients [189]. Dysgeusia occurred in approximately half of treated patients, with additional gastrointestinal and bone-related adverse events reported. ETC-159 likewise produced dose-dependent pharmacodynamic inhibition of Wnt signaling but was associated with increased β-CTX, a marker of bone resorption, and decreased bone mineral density in some patients. Thus, systemic drug exposure and measurable target engagement have not consistently been translated into meaningful antitumor activity. Combination strategies have also been explored, including WNT974 with encorafenib and cetuximab in BRAF V600E colorectal cancer; however, the development of this regimen was discontinued after limited evidence of incremental efficacy together with continued safety concerns, including bone toxicity [190].

#### 4.6.3. β-Catenin Inhibition and Modulation Limitations

These considerations are particularly relevant to HCC, which frequently develops in the setting of cirrhosis and impaired hepatic reserve. Drug-induced hepatotoxicity or alterations in bilirubin metabolism may therefore have greater clinical consequences in this population. Reversible grade 3 hyperbilirubinemia was observed with PRI-724 in a phase I solid-tumor study, while reversible hyperbilirubinemia and anemia have been reported in the ongoing clinical evaluation of tegavivint in HCC [191]. E7386 has been associated with nausea and vomiting as well as substantial interpatient pharmacokinetic variability. Accordingly, dose optimization in HCC requires consideration of both systemic pathway-related toxicity and the reduced physiologic reserve associated with chronic liver disease.

Pharmacokinetic and drug-delivery limitations vary according to therapeutic modality. Early β-catenin/TCF inhibitors were limited by suboptimal potency, selectivity, and pharmacokinetic properties. PRI-724 requires continuous intravenous infusion, whereas peptide-based BCL9 inhibitors face challenges related to stability, tissue penetration, and intracellular delivery [155,191]. Nucleic acid-based approaches provide an alternative means of overcoming the structural limitations of β-catenin as a conventional drug target but introduce additional challenges related to tumor-selective delivery, endosomal escape, durability of target suppression, and potential innate immune activation. Heterogeneous intratumoral distribution may further result in incomplete target suppression and permit persistence, or re-emergence of β-catenin-active tumor subclones [192].

An additional challenge arises from the signaling-independent functions of β-catenin at adherens junctions, where it contributes to epithelial integrity. Global β-catenin depletion may therefore disrupt normal cell–cell adhesion in addition to suppressing oncogenic transcription. Strategies that preferentially target the transcriptionally active nuclear pool of β-catenin through cofactors such as TBL1, CBP, or BCL9 may provide a more favorable therapeutic window than indiscriminate pathway inhibition. Early clinical activity observed with tegavivint in biomarker-selected HCC provides preliminary support for this approach, although further validation is required. More broadly, the limited efficacy and mechanism-based toxicity observed with earlier Wnt-pathway inhibitors indicate that successful clinical development will likely require molecular patient selection, tumor-selective delivery, and rational combination strategies rather than uniform systemic suppression of Wnt/β-catenin signaling.

## 5. Future Directions/Conclusions

Several fundamental questions remain regarding the role of β-catenin in tumorigenesis, progression and therapeutic intervention of HCC. Further clarification as to the differential biology of the gain-of-function *CTNNB1* and loss-of-function AXIN1 mutation is necessary as genetic studies have found these two mutations to be mutually exclusive within the HCC population. The *CTNNB1* mutation is often associated with the non-proliferative group and relies on canonical β-catenin signaling, while the AXIN1 mutations often belong to the proliferative group and rely on YAP/NOTCH induction rather than β-catenin activation [48,193,194]. Understanding these subclasses and the roles these mutations play is essential in determining the therapeutic strategy necessary for patients demonstrating this mutation. Additionally, understanding the role of Wnt ligands in *CTNNB1* mutant versus wild-type HCC tumors would provide some clarity into whether therapies targeting Wnt ligands might remain effective in these mutant populations [66]. Patients with Wnt/β-catenin activation, specifically those with *CTNNB1* mutations, have been found to have a worse response to immunotherapies [147]. Interestingly, MASH-related HCCs have also been found to be particularly resistant to immunotherapies. Since the status of β-catenin in these cases has not been well characterized, it brings to question if this pathway plays a critical role in this poor response [195]. Although approaches such as gene editing and nucleic-acid based therapies for the major mutation *CTNNB1* or the gene as a whole are conceptually attractive, delivery challenges and the potential that wild-type β-catenin is sufficient to support HCC progression remains a substantial hurdle for this class of therapeutics. Finally, due to the essential role β-catenin plays in intestinal stem cell renewal, systemic inhibition results in significant gastrointestinal adverse effects, motivating the development of tumor-selective delivery systems and mutation-specific targeting strategies.

Aberrant activation of β-catenin signaling is one of the frequent molecular events in HCC, occurring in nearly a third of cases and driving a distinct tumor phenotype characterized by cancer stemness, immune exclusion, metabolic reprogramming, and broad-spectrum therapeutic resistance. The mechanisms linking β-catenin to therapeutic resistance are becoming increasingly well defined, and the pathways crosstalk with other oncogenic pathways, such as PI3K/AKT, RAS/MAPK, and YAP signaling, highlighting the necessity for a combination approach to therapeutic intervention. β-catenin was historically considered undruggable due to its structural featurelessness, essential physiological function and on-target toxicity, and multiple mutant forms rendering upstream targeting relatively ineffective. β-catenin’s structure is rigid and flat with no deep hydrophobic grooves or pockets that could be utilized for conventional drug target design [196]. It is also a ubiquitously expressed protein with many essential cellular roles; therefore, systemic inhibition would result in severe toxicity in normal tissues. Finally, mutations in β-catenin can render it constitutively active and impervious to degradation, making any upstream drug targets ineffective against these mutant forms [197]. This resulted in a shift in the therapeutic landscape focusing on the use of specialized drug classes that overcome these limitations. Helicons (alpha-helically constrained polypeptides) are synthetically stabilized peptides that are able to bind to proteins with large, flat protein surfaces, like β-catenin [198]. Screening of Helicon libraries identified multiple binding families for β-catenin. PROTACs (Proteolysis-Targeting Chimeras) could resolve the prior limitation of β-catenin escaping degradation. These molecules are able to bind to protein targets and recruit E3 ubiquitin ligases, marking the protein for proteasomal degradation [199]. A class of Helicons has even been developed that is able to simultaneously bind to β-catenin and E3 ubiquitin ligases, inducing a cooperative ternary complex [200]. While these new therapeutic methods better address the drugability of β-catenin, they still result in off-target effects; therefore, the development of tumor- and mutation-specific therapeutic options would eliminate this limitation. The convergence of these new therapeutic options, deep molecular stratification of tumors, and increased mechanistic understanding of resistance has positioned β-catenin as one of the most promising precision targets in advanced HCC. The path forward lies in biomarker-enriched clinical trials that pair these emerging therapies with the current standards in the field tailored specifically for the molecular context of each patient’s tumor.

## Figures and Tables

**Figure 1:**
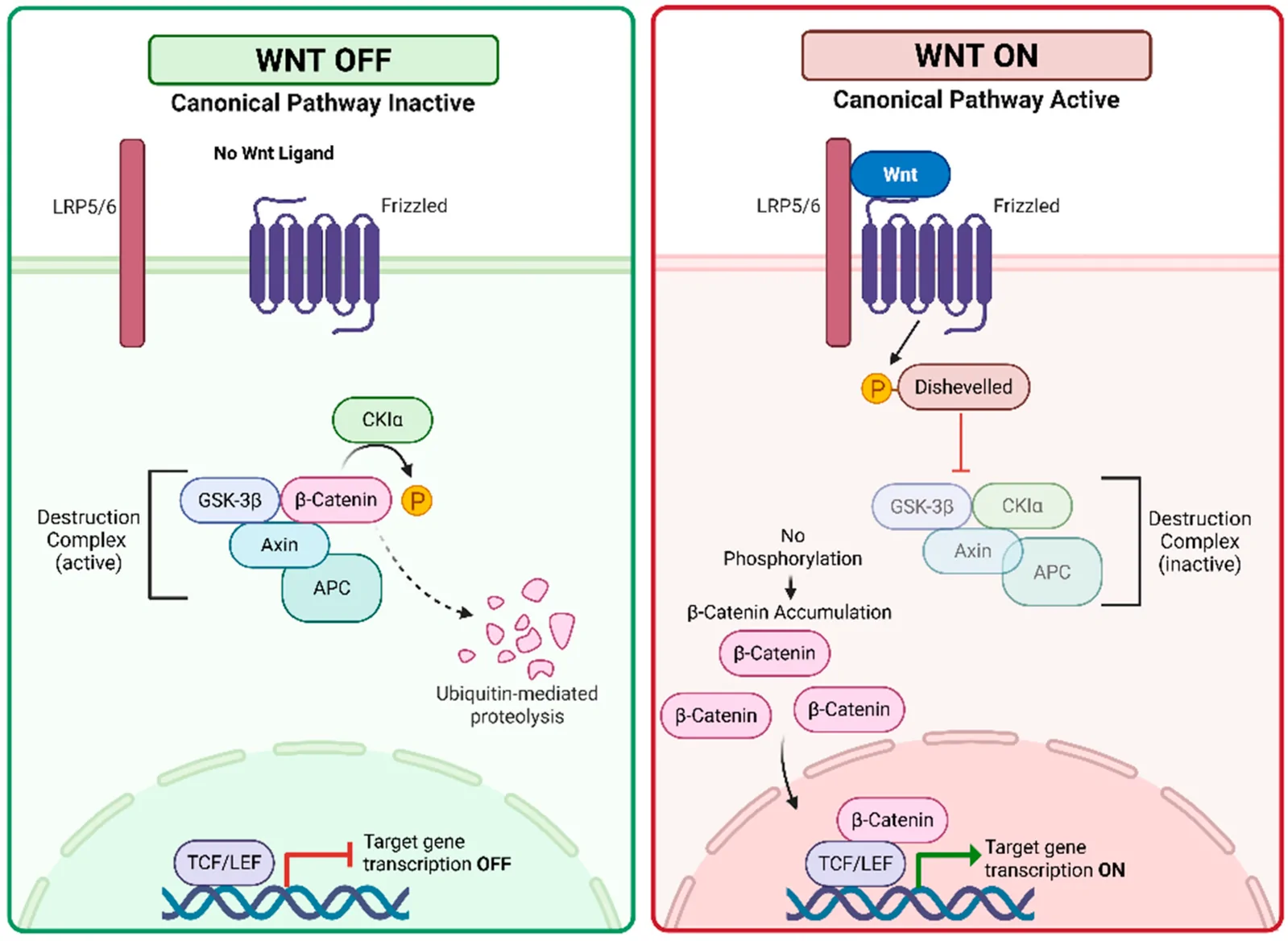
Canonical Wnt/β-catenin signaling in normal cells: off and on states. The absence of Wnt ligand (**left**) results in continuous phosphorylation of β-catenin by the destruction complex (APC, Axin, GSK-3β, and CK1α), which leads to ubiquitin-mediated proteolysis of β-catenin. Low cytoplasmic β-catenin levels due to degradation prevent nuclear translocation and subsequent activation of Wnt target genes, resulting in the Wnt off state. In the presence of the Wnt ligand (**right**), Wnt binds to Frizzled and LRP5/6, which then inhibit the destruction complex, allowing β-catenin to accumulate in the cytoplasm. The cytoplasmic β-catenin translocates to the nucleus where it interacts with TCF/LEF transcription factors to activate Wnt target gene transcription, resulting in the Wnt on state. Black arrows indicate something acting on or resulting in an action, red indicates inhibition, and green indicates activation. APC, adenomatous polyposis coli; CK1α, Casein Kinase 1α; GSK-3β, Glycogen Synthase Kinase-3β; LRP5/6, Low-Density Lipoprotein Receptor-Related Proteins 5 and 6; TCF/LEF, T-cell factor/Lymphoid Enhancer-Binding Factor.

**Figure 2:**
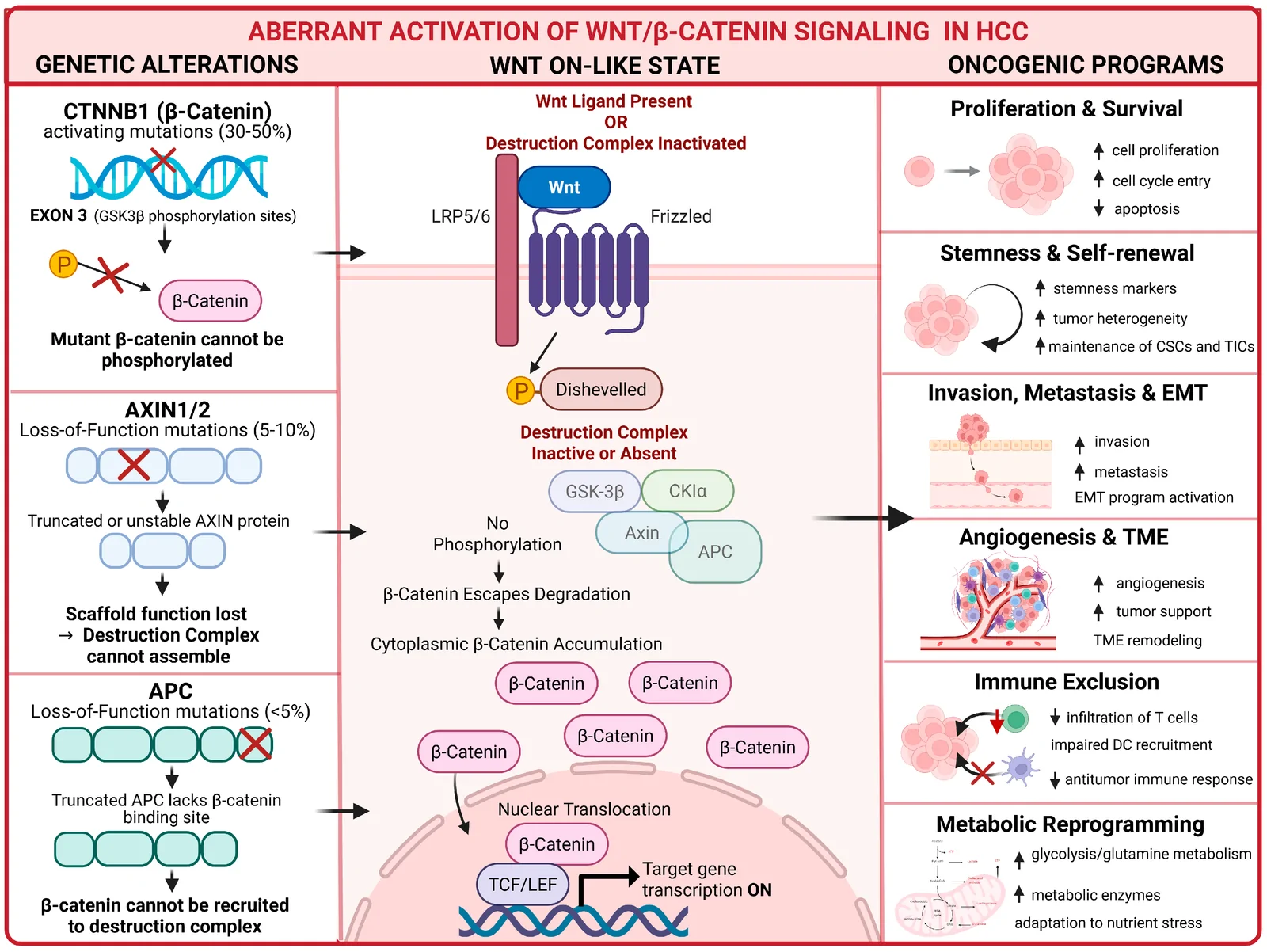
Aberrant activation of Wnt/β-catenin signaling in HCC. Genetic alteration to *CTNNB1* (β-catenin gene) or components of the destruction complex (AXIN1/2 and APC) prevent the phosphorylation and degradation of β-catenin, resulting in activation of Wnt target gene transcription and subsequent initiation of oncogenic programs, such as proliferation and survival, stemness and self-renewal, invasion, metastasis, and EMT, angiogenesis and TME, immune exclusion, and metabolic reprogramming. Black arrows indicate something acting on or resulting in an action, red indicates inhibition or deletion, and upward/downward facing arrow indicates and increase/decrease respectively. APC, adenomatous polyposis coli; AXIN, axis inhibition; CK1α, Casein Kinase 1α; EMT, epithelial–mesenchymal transition; GSK-3β, Glycogen Synthase Kinase-3β; LRP5/6, Low-Density Lipoprotein Receptor-Related Proteins 5 and 6; TCF/LEF, T-cell factor/Lymphoid Enhancer-Binding Factor; TME, tumor microenvironment.

**Figure 3:**
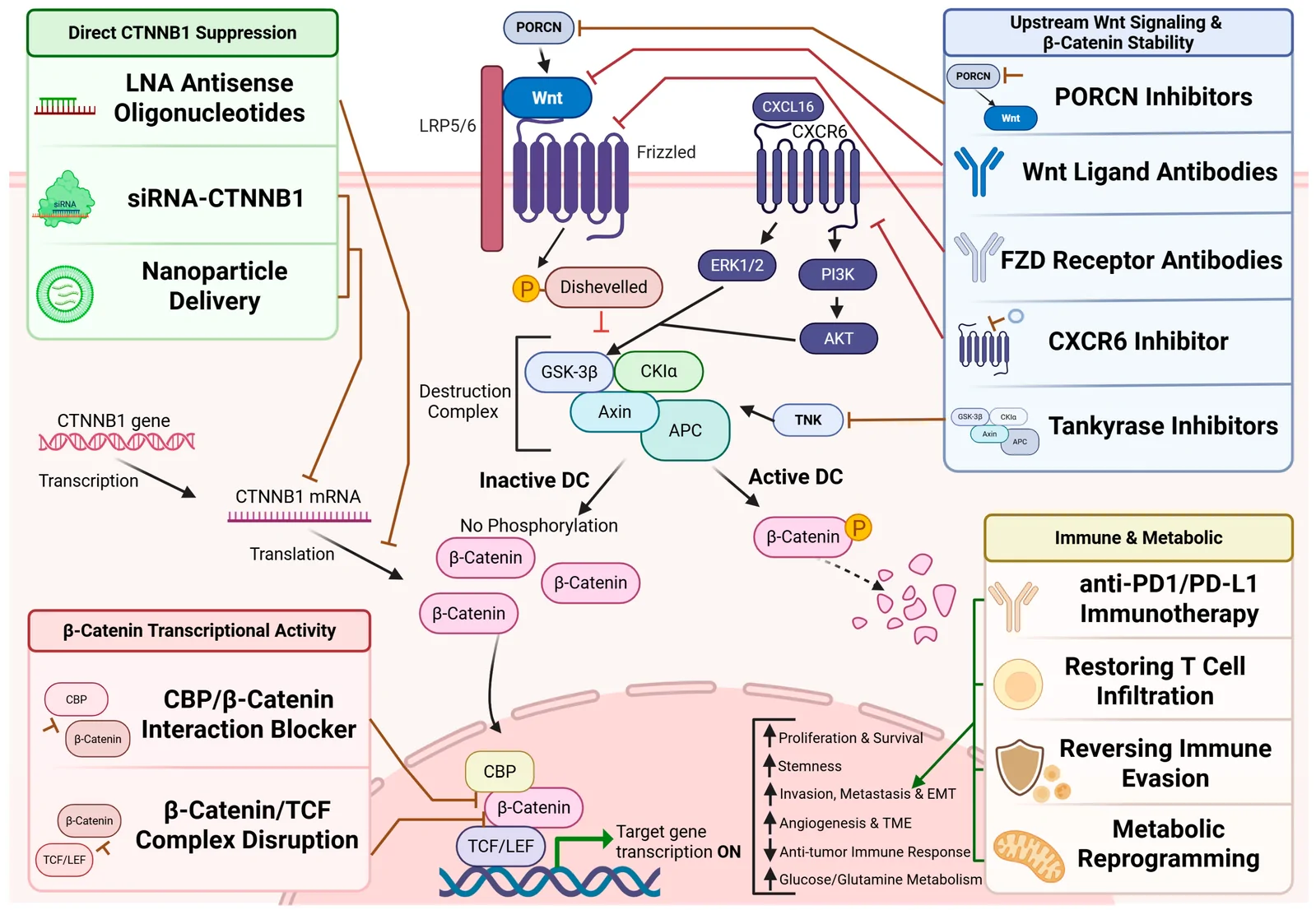
Therapeutic strategies targeting β-catenin in HCC. This figure demonstrates the mechanisms by which the four major categories of therapeutic strategies for targeting Wnt/β-catenin signaling function. The four strategies include: β-catenin transcriptional activity via CBP and TCF interactions with β-catenin, upstream Wnt signaling and β-catenin stability via Wnt or Frizzled antibodies and PORCN, CXCR6, or Tankyrase inhibition, direct *CTNNB1* suppression via antisense-mediated CTNNB1 suppression and RNA interference, and immune and metabolic reprogramming via anti-PD1/PD-L1 immunotherapy, restoring T cell infiltration, reversing immune evasion, and metabolic reprogramming. Red lines indicate inhibition, green arrows indicate activation, and black indicates the flow of the pathway and direction of action. APC, adenomatous polyposis coli; AXIN, axis inhibition; CBP, CREB-binding protein; CK1α, Casein Kinase 1α; CXCR6, C-X-C Chemokine Receptor 6; EMT, epithelial–mesenchymal transition; FZD, Frizzled; GSK-3β, Glycogen Synthase Kinase-3β; LRP5/6, Low-Density Lipoprotein Receptor-Related Proteins 5 and 6; PD1/PD-L1, Programmed Cell Death Protein 1/Programmed Death Ligand 1; PORCN, Porcupine O-Acyltransferase; TCF/LEF, T-cell factor/Lymphoid Enhancer-Binding Factor; TME, tumor microenvironment; TNK, Tankyrase.

**Table 1:** Major therapeutic strategies targeting Wnt/β-catenin signaling.

Category	Therapeutic Strategy	Representative Agent(s)	Mechanism	Potential Advantages	Major Limitations	Development Status/Clinical Findings
β-Catenin Transcriptional Activity	β-Catenin–TCF/LEF disruption	PKF115-854; CGP049090	Disrupts β-catenin–TCF/LEF interaction and suppresses canonical transcription	Directly targets downstream transcriptional output; established proof of principle for pharmacologic β-catenin inhibition	Limited potency/selectivity; off-target effects; no successful clinical development	Preclinical
	Direct β-catenin–TCF inhibition	FOG-001 (zolucatetide)	Directly disrupts β-catenin–TCF transcriptional complex	Acts downstream of ligand/receptor signaling; potentially relevant to tumors with CTNNB1 or other downstream Wnt alterations	Therapeutic window, optimal dose and durability remain undefined; mature HCC efficacy data unavailable	Phase I/II, NCT05919264; ongoing. Dedicated HCC cohort included
	β-Catenin–CBP inhibition	ICG-001; PRI-724	Disrupts β-catenin interaction with CBP and alters oncogenic transcription	More selective transcriptional modulation than global β-catenin depletion; may enhance sorafenib-induced apoptosis	Limited monotherapy activity; PRI-724 requires prolonged IV infusion; PK constraints	Early phase clinical development. PRI-724 showed pharmacodynamic activity but limited monotherapy efficacy
	Oral β-catenin/CBP-directed modulation	E7386	Inhibits β-catenin/CBP-associated transcription	Oral administration; downstream strategy applicable to CTNNB1-mutant disease; amenable to TKI/ICI combinations	Limited monotherapy activity; nausea/vomiting; interpatient PK variability; biomarker population unresolved	Phase I/Ib-II. No objective responses with monotherapy in CTNNB1-mutant HCC expansion; combination development with lenvatinib and pembrolizumab
	BCL9 Cofactor inhibition	hsBCL9Z96	Disrupts BCL9-dependent β-catenin transcriptional activity	May inhibit tumor signaling while restoring antigen presentation, macrophage function and CD8^+^ infiltration; synergizes with anti-PD-L1 in models	Peptide stability, tissue penetration and intracellular delivery; no clinical validation	Preclinical
	TBL1/β-catenin inhibition	Tegavivint (BC2059)	Binds TBL1 and disrupts the TBL1–β-catenin complex, preferentially reducing transcriptionally active nuclear β-catenin	Downstream approach may overcome ligand independence; may spare membrane/cytoplasmic β-catenin; biomarker-selected activity reported	IV administration; anemia and hyperbilirubinemia; evidence remains preliminary	Phase I/II, NCT05797805; ongoing. Preliminary activity concentrated in Wnt-pathway-altered HCC
Upstream Wnt Signaling & β-Catenin Stability	Wnt-ligand neutralization	Anti-Wnt2 antibodies	Neutralizes extracellular Wnt ligand and prevents receptor activation	Extracellular, potentially selective strategy for ligand-dependent tumors	Pathway redundancy; unlikely to overcome constitutive downstream CTNNB1 activation	Preclinical in HCC
	Frizzled receptor blockade	Vantictumab (OMP-18R5)	Antibody against FZD1/2/5/7/8 prevents Wnt–Frizzled signaling	Broad extracellular inhibition of ligand-dependent canonical Wnt signaling	Fragility fractures and skeletal toxicity; narrow therapeutic index; ineffective against ligand-independent downstream activation	Phase Ib development discontinued/limited. Bone-related toxicity constrained further development
	Wnt-ligand sequestration	Ipafricept (OMP-54F28)	Soluble FZD8-Fc decoy receptor sequesters extracellular Wnt ligands	Broad ligand sequestration; potential combination strategy in ligand-dependent disease	Bone-resorption changes and fragility fractures; ineffective in ligand-independent tumors	Phase Ib HCC trial completed (NCT02069145). HCC efficacy results not posted; broader development limited by bone toxicity
	PORCN inhibition	WNT974 (LGK974)	Inhibits PORCN-mediated Wnt palmitoylation and secretion	Oral therapy; measurable AXIN2 target engagement; rational in ligand-dependent Wnt tumors	Limited activity in CTNNB1/APC-driven disease; dysgeusia and skeletal/GI toxicity; target engagement without robust tumor responses	Phase I completed. No RECIST responses in first-in-human study; SD in a minority of patients
	PORCN inhibition ± ICI	ETC-159 (ETC-1922159)	Inhibits PORCN and Wnt ligand secretion	Oral drug; strong AXIN2 suppression; combination with ICI is mechanistically attractive	Ligand dependence; β-CTX elevation and reduced BMD; limited monotherapy efficacy	Phase Ia/Ib. Monotherapy: no objective responses; ETC-159 + pembrolizumab: 1 PR and 5 SD among 15 evaluable patients
	Destruction-complex stabilization	XAV939; G007-LK; JW55	Tankyrase inhibition stabilizes AXIN and enhances β-catenin degradation	Restores endogenous destruction-complex activity	May not overcome degradation-resistant CTNNB1 mutations; normal-tissue Wnt effects possible; no established HCC clinical data	Preclinical in HCC
	CXCR6 inhibition	SBI-457	Reduces CXCR6-associated p38 signaling, GSK3β Ser9 phosphorylation and β-catenin stabilization	May modulate resistance-associated β-catenin signaling without global Wnt inhibition; may attenuate sorafenib-associated pathway activation	Indirect mechanism; evidence largely from cell and xenograft models	Preclinical
	Indirect/repurposed pathway modulation	Aspirin, indomethacin, sulindac, celecoxib; astaxanthin	Indirect COX-dependent/independent Wnt modulation or promotion of β-catenin degradation	Existing pharmacology for some agents; potential adjunctive approaches	Nonspecific effects; modest activity; uncertain degree of pathway inhibition; no validated β-catenin-directed role in HCC	Preclinical/exploratory
Direct CTNNB1 Suppression	Antisense-mediated CTNNB1 suppression	LNA antisense oligonucleotides	Reduces CTNNB1 mRNA and β-catenin protein expression	Acts downstream of Wnt ligand/receptor signaling; potentially applicable to CTNNB1-driven HCC	Delivery and tissue penetration; suppression of physiologic β-catenin; durability and systemic toxicity unresolved	Preclinical. Reduced tumor growth in CTNNB1-mutant models; activity appears genotype-dependent
	RNA interference	CTNNB1 siRNA	Sequence-specific degradation of CTNNB1 mRNA	Direct targeting of mutation-driven downstream signaling; avoids requirement for a conventional β-catenin-binding pocket	RNA instability, intracellular delivery and endosomal escape; potential innate immune activation	Preclinical
	Nanoparticle-mediated RNA interference	LNP-CTNNB1 siRNA	Lipid nanoparticles deliver CTNNB1-directed siRNA to tumor cells	Strong antitumor activity in CTNNB1-mutant models; can remodel metabolic state, restore antigen presentation and enhance ICI response	Tumor-selective biodistribution, heterogeneous intratumoral exposure, endosomal escape and durability of knockdown; no established human HCC efficacy	Preclinical. Tumor regression, metabolic reprogramming and reversal of immune exclusion demonstrated in HCC models
Immune & Metabolic	Combination with immune checkpoint blockade	Anti-PD-1/anti-PD-L1 + β-catenin- directed therapy	β-Catenin inhibition may restore inflammatory signaling, dendritic-cell/T-cell recruitment and responsiveness to ICI	Addresses both oncogenic signaling and immune exclusion; strong mechanistic rationale	Most combination evidence remains preclinical or early phase; appropriate biomarker population not yet established	Preclinical to early clinical. E7386, ETC-159 and tegavivint combination strategies under clinical investigation
	Restoration of T-cell infiltration/immune reprogramming	CTNNB1 siRNA; BCL9 inhibition; CBP inhibition	Restores interferon-associated signaling, antigen presentation and CD8^+^ T-cell recruitment	May convert immune-excluded β-catenin-active HCC to a more immune-responsive phenotype	Clinical relevance of immune reprogramming remains unproven; effects may be context dependent	Predominantly preclinical
	Metabolic reprogramming	CTNNB1 suppression	Disrupts β-catenin-dependent pericentral/zone 3 metabolic programs and shifts tumor phenotype toward zone 1/2-like state	Potentially targets a defining metabolic dependency of CTNNB1-mutant HCC; may complement targeted or immune therapy	Metabolic consequences in patients remain undefined; no validated metabolic companion biomarker	Preclinical

This table summarizes the four major categories of therapeutic strategies for targeting Wnt/β-catenin signaling, including representative therapeutic agents for each strategy and their specific mechanisms. The four major categories addressed in this table include: β-catenin transcriptional activity, upstream Wnt signaling and β-catenin stability, direct CTNNB1 suppression, and immune and metabolic strategies. For each therapeutic agent, the table also compares potential advantages and major limitations, along with the current clinical findings/developments discussed in the literature. APC, adenomatous polyposis coli; BCL9, B-cell Lymphoma 9; CBP, CREB-binding protein; COX, cyclooxygenase; CXCR6, C-X-C Chemokine Receptor 6; HCC, hepatocellular carcinoma; ICI, immune checkpoint inhibitor; LEF, Lymphoid Enhancer-Binding Factor; LNP, lipid nanoparticle; PD-1, Programmed Cell Death Protein 1; PD-L1, Programmed Death Ligand 1; PORCN, Porcupine O-Acyltransferase; TBL1, Transducin B-Like Protein 1; TCF, T-cell factor; TKI, tyrosine kinase inhibitor.

**Table 2:** Clinical development and translational limitations of representative Wnt/β-catenin-directed therapies.

Agent	Target/Level of Inhibition	Clinical Setting	Trial/Status	Major Findings	Principal Limitation/Toxicity
Vantictumab (OMP-18R5)	FZD1/2/5/7/8 receptor blockade	Metastatic pancreatic cancer + gemcitabine/nab-paclitaxel	Phase Ib; terminated because of bone-safety concerns	Fragility fractures occurred at 7 mg/kg; dosing schedule had to be modified	On-target skeletal toxicity and narrow therapeutic window
Vantictumab	Frizzled receptor blockade	HER2-negative breast cancer + paclitaxel	Phase Ib; completed	ORR 31.3%; six patients developed fractures	Fracture incidence limited further clinical development
Ipafricept (OMP-54F28)	FZD8-Fc decoy receptor; Wnt-ligand sequestration	Advanced solid tumors	Phase I; completed	Target/pathway modulation; prolonged SD in selected patients; fragility fractures at 20 mg/kg	Bone-resorption changes and fragility fractures at high exposure
Ipafricept + sorafenib	Wnt-ligand sequestration + multikinase inhibition	HCC	NCT02069145; completed	Dedicated HCC phase Ib trial	Mature peer-reviewed efficacy results have not been identified; clinical benefit remains uncertain
WNT974/LGK974	PORCN inhibition; blocks Wnt secretion	Wnt-dependent advanced solid tumors	NCT01351103; Phase I; completed	*n* = 94; 0 objective responses; SD 16%; AXIN2 suppression confirmed pharmacodynamic activity	Dysgeusia 50%; pathway inhibition did not translate into substantial monotherapy efficacy
WNT974 + encorafenib + cetuximab	PORCN + BRAF + EGFR inhibition	BRAF V600E metastatic CRC	NCT02278133; Phase Ib/II; discontinued	ORR 10%; DCR 85%	Bone toxicities in 9/20 patients; insufficient evidence of added efficacy; safety and efficacy concerns caused discontinuation
PRI-724	CBP–β-catenin interaction inhibition	Advanced solid tumors ± mFOLFOX6	NCT01302405; Phase Ia/Ib; terminated	Grade 3 hyperbilirubinemia DLT; pharmacodynamic survivin suppression	Seven-day continuous IV infusion; PK plateau at higher doses; trial terminated for low enrollment, not toxicity
E7386 monotherapy	β-catenin–CBP-directed protein–protein interaction inhibitor	Advanced tumors; CTNNB1-mutant HCC expansion	NCT03264664; Phase I; active, not recruiting	No objective responses; SD 36.8% overall and 2/6 CTNNB1-mutant HCC patients	Nausea/vomiting common; marked interpatient PK variability; limited monotherapy efficacy
E7386 + lenvatinib	β-catenin-directed modulation + VEGFR/FGFR multikinase inhibition	Advanced HCC	NCT04008797; Phase I/II; active, not recruiting	Translational cohort: 3/7 patients showed ≥30% maximum tumor-diameter reduction; ATF4/antiangiogenic effects observed	Small clinical dataset and no definitive comparative efficacy yet
E7386 + pembrolizumab ± lenvatinib	β-catenin modulation + checkpoint inhibition ± TKI	Previously treated HCC, melanoma and CRC	NCT05091346; Phase Ib/II; completed	89 patients enrolled; registry results posted	Full HCC-specific peer-reviewed efficacy publication remains limited
Tegavivint (BC2059)	TBL1 inhibition; disrupts nuclear β-catenin transcriptional signaling	Advanced HCC	NCT05797805; Phase I/II; ongoing	2026 dose-finding data: in molecularly selected Wnt-mutant patients treated within recommended dose range and ≤3 previous lines, ORR 22%, DCR 89%, median PFS 8 months	Grade 3 anemia at higher dose; reversible hyperbilirubinemia and anemia; results remain preliminary
FOG-001/zolucatetide	Direct β-catenin–TCF interaction inhibitor	Wnt-pathway-activated solid tumors, including HCC cohort	NCT05919264; Phase I/II; recruiting	Direct downstream β-catenin strategy; HCC cohort included	HCC efficacy and therapeutic window remain investigational; no mature HCC peer-reviewed dataset
ETC-159/ETC-1922159	PORCN inhibition	Advanced solid tumors ± pembrolizumab	NCT02521844; Phase I; active, not recruiting	Demonstrates continued clinical investigation of ligand-dependent Wnt blockade	Expected biological limitation in tumors with downstream CTNNB1/APC activation

This table provides a detailed review of representative therapeutic agents targeting Wnt/β-catenin signaling, focusing on their clinical development and application. The table includes general details regarding the target/level of inhibition, clinical setting, and trial/status associated with each agent. It examines major findings and significant limitations/toxicities identified in both the literature and clinical trials relevant to the respective agents. AXIN2, Axis Inhibition 2; CBP, CREB-binding protein; FZD1, Frizzled 1; HCC, hepatocellular carcinoma; PFS, progression-free survival; PORCN, Porcupine O-Acyltransferase; TBL1, Transducin B-Like Protein 1; TCF, T-cell factor; VEGFR, Vascular Endothelial Growth Factor Receptor.

## Data Availability

No new data were created or analyzed in this study. Data sharing is not applicable to this article.

## Abbreviations

The following abbreviations are used in this manuscript

ABC	ATP-Binding Cassette
AFP	Alpha-fetoprotein
AKT	Protein Kinase B
APC	Adenomatous Polyposis Coli
AXIN	Axis Inhibition
BCL9	B-cell Lymphoma 9
β-TrCP	β-Transducin Repeat-Containing Protein
CAR	Chimeric Antigen Receptor
CBP	CREB-Binding Protein
CCA	Cholangiocarcinoma
CCL5	Chemokine (C-C Motif) Ligand 5
CD24	Cluster of Differentiation 24
CD133	Prominin-1
CK1	Casein Kinase 1
COX	Cyclooxygenase
CSCs	Cancer Stem Cells
CTLA-4	Cytotoxic T-Lymphocyte-Associated Protein 4
CXCL16	C-X-C Chemokine Ligand 16
CXCR6	C-X-C Chemokine Receptor 6
EMT	Epithelial–Mesenchymal Transition
EpCAM	Epithelial Cell Adhesion Molecule
EPHB2	Ephrin Type-B Receptor 2
ERK	Extracellular Signal-Regulated Kinase
FZD	Frizzled
GSK3β	Glycogen Synthase Kinase-3β
HAIC	Hepatic Arterial Infusion Chemotherapy
HCC	Hepatocellular Carcinoma
HIF-1α	Hypoxia-Inducible Factor-1α
HNF4α	Hepatocyte Nuclear Factor 4α
ICIs	Immune Checkpoint Inhibitors
IFN-α	Interferon-α
IL-6	Interleukin-6
IRF	Interferon Regulatory Factor
IRF2	Interferon Regulatory Factor 2
JAK	Janus Kinase
LEF	Lymphoid Enhancer-Binding Factor
LGR5	Leucine-Rich Repeat-Containing G-Protein-Coupled Receptor 5
LNP	Lipid Nanoparticle
LRP5/6	Low-Density Lipoprotein Receptor-Related Proteins 5 and 6
LRP8	Low-Density Lipoprotein Receptor-Related Protein 8
LSD1 (KDM1A)	Lysine-Specific Demethylase 1
MAFLD	Metabolic Dysfunction-Associated Fatty Liver Disease
MAPK	Mitogen-Activated Protein Kinase
MASH	Metabolically Associated Steatohepatitis
MEK	Mitogen-Activated Protein Kinase Kinase
MET	Mesenchymal–Epithelial Transition factor
METTL3	Methyltransferase-Like 3
mTOR	Mammalian Target of Rapamycin
MYC	Myelocytomatosis Oncogene
NEK2	NIMA-Related Kinase 2
NF-κB	Nuclear Factor Kappa-Light-Chain-Enhancer of Activated B Cells
NRF2	Nuclear Factor Erythroid 2-Related Factor 2
OS	Overall Survival
PD-1	Programmed Cell Death Protein 1
PD-L1	Programmed Death Ligand 1
PFS	Progression-Free Survival
PI3K	Phosphoinositide 3-Kinase
PORCN	Porcupine O-Acyltransferase
POU2F1	POU Class 2 Homeobox 1
PPAR	Peroxisome Proliferator-Activated Receptor
PROX1	Prospero-Related Homeobox 1
RAF	Rapidly Accelerated Fibrosarcoma
RAB27A	Ras-Related Protein Rab-27A
RAS	Rat Sarcoma
ROS	Reactive Oxygen Species
R-Spo1	R-Spondin-1
RUNX3	Runt-Related Transcription Factor 3
SDC4	Syndecan-4
SIGLEC10	Sialic Acid-Binding Ig-Like Lectin 10
siRNA	Small Interfering RNA
SRC	Proto-Oncogene Tyrosine-Protein Kinase Src
STAT3	Signal Transducer and Activator of Transcription 3
TACE	Transarterial Chemoembolization
TBL	Transducin β-Like Protein
TCF	T-Cell Factor
TGF-β	Transforming Growth Factor-β
TICs	Tumor-Initiating Cells
TKI	Tyrosine Kinase Inhibitor
TNFα	Tumor Necrosis Factor-α
VEGF	Vascular Endothelial Growth Factor
VEGFR	Vascular Endothelial Growth Factor Receptor
Wnt	Wingless-Related Integration Site
YAP	Yes-Associated Protein
ZEB1	Zinc Finger E-Box Binding Homeobox 1
5-FU	5-Fluorouracil
